# Three birds with one stone: oxygen self-supply engineering palladium nanocluster/titanium carbide hybrid for single-NIR laser-triggered synergistic photodynamic-photothermal therapy

**DOI:** 10.1515/nanoph-2022-0268

**Published:** 2022-08-05

**Authors:** Shanshan Dang, Yanmei Mo, Junqing Zeng, Yunjie Xu, Zhongjian Xie, Han Zhang, Bin Zhang, Guohui Nie

**Affiliations:** Shenzhen Key Laboratory of Nanozymes and Translational Cancer Research, Institute of Translational Medicine, Department of Otolaryngology, Shenzhen Second People’s Hospital, the First Affiliated Hospital of Shenzhen University, Health Science Center, Institute of Microscale Optoelectronics, Shenzhen 518035, China,718426557@qq.com 2807751582@qq.com xuyunjie87@163.com zjxie2011@163.com; Graduate Collaborative Training Base of Shenzhen Second’ People’s Hospital, Hengyang Medical School, University of South China, Hengyang, Hunan, 421001, China, doczgjnqi@126.com

**Keywords:** 2D nanosheets, enhanced photodynamic therapy, molecular oxygen, photothermal therapy, reactive oxygen species

## Abstract

As a key branch of the cross-discipline biophotonics, phototherapy, including photodynamic therapy (PDT), and photothermal therapy (PTT), is promising in biomedicine and visible light-driving PDT has been applied to clinical treatment. However, extensive applications of phototherapy are limited by the hypoxic microenvironment, laser penetration depth, and potential complexity for combined PDT/PTT. Thus, NIR-responsive oxygen self-supply nanocomposites functionalized with photosensitizers for achieving simultaneous in-depth PDT/PTT are urgently required. Herein, a multifunctional platform has been fabricated by co-immobilizing monodispersed ultrasmall Pd nanoclusters and a photosensitizer 5,10,15,20-Tetrakis (4-Aminophenyl)-21H,23H Porphyrin (Thp) on the surface of Ti_3_C_2_T_*x*_ MXene nanosheets, generating the Pd-Thp-Ti_3_C_2_T_*x*_ nanocomposite. Material characterization demonstrated that Pd nanoclusters and Thp were well-distributed on the MXene surface while MXene maintained its photothermal conversion efficiency and broad absorption. In this nanoplatform, irradiated by the single 808 nm laser, Pd selectively catalyzed the decomposition of H_2_O_2_ to O_2_, and O_2_ was continuously supplied to Thp for enhanced NIR-driving PDT. The *in vivo* fluorescence and photothermal imaging demonstrated the pronounced accumulation of nanocomposites in the tumor site. Both *in vitro* and *in vivo* results clearly demonstrated the nanocomposite had good biocompatibility, and that the synergistic PTT and enhanced PDT made apoptosis of the tumor cell achievable. This work not only proves this Pd-Thp-Ti_3_C_2_T_*x*_ nanocomposite serves a promising solution for tumor hypoxia by inducing apoptosis of tumor cells with synergistic PTT and PDT, but also broadens the application of promising optical materials in biomedical field.

## 1 Introduction

Over the years, the morbidity and fatality rate of malignant tumors has been rising, with serious threatens to human life and health [1]. Traditional tumor therapy (such as surgery, chemotherapy, and radiotherapy) usually has side effects like low therapeutic efficiency, invasiveness and limited inhibition of tumor due to drug resistance. Therefore, it is necessary to develop new tumor treatment methods with high efficiency and minimal side effects. With the continuous development of photosensitive materials and nanotechnology, phototherapy provides a minimally invasive, highly effective, and smart switch-off therapeutic approach for tumor treatment, which generally includes photothermal therapy (PTT) and photodynamic therapy (PDT) [2], [3], [4], [5], [6]. PTT is generally based on heat generated from light energy absorbed by photothermal conversion agents, causing irreversible thermal damage to tumor tissues, and thus, leading to tumor ablation. However, PTT usually needs high-power and long-time irradiation to generate enough heat to kill cells, which may decrease the therapeutic effect or cause local overheating. PDT utilizes the reaction between photosensitizer with oxygen (O_2_) or hydrogen peroxide (H_2_O_2_) under certain laser irradiation to produce reactive oxygen species (ROS), like singlet oxygen (^1^O_2_) and hydroxyl radical (•OH), triggering tumor cell apoptosis through oxidative stress. However, the effect might be restricted because of the hypoxic nature of solid tumors. Moreover, the hypoxic environment would further affect the cellular expression program and lead to therapy resistance. Although Type I PDT, which converts H_2_O_2_ to •OH, might offer a solution for hypoxia issue, H_2_O_2_ thermodynamically prefers to decompose to H_2_O and O_2_ with low energy barrier. The result of such competitive conversion of H_2_O_2_ may decrease the efficiency of PDT. Thus, there is a need for the rational design of materials that can selectively catalyze the decomposition of H_2_O_2_ into O_2_ and stimulate type II PDT (O_2_ to ^1^O_2_) process, which may help solve the PDT issue.

Currently, various strategies with desired synergistic effect of PTT and PDT have been developed for cancer phototherapy with the potential for clinical applications [7], [8], [9], [10], [11]. However, there are still some defects such as, the excitation of photosensitizer used in traditional PDT need the ultraviolet light or visible light, which has limited penetration and damages normal cells when exposed for a long time [12, 13]. Therefore, the use of short wavelength excitation in PDT treatment has significant disadvantages. Recently, two-dimensional (2D) materials, including graphene and black phosphorus nanosheets [14], [15], [16], [17], [18], were found to exhibit perfect effect of phototherapy due to their unique physicochemical properties and photothermal conversion efficiency under the irradiation of near-infrared (NIR) laser. As the latest emerging 2D materials, MXenes have attracted more attention in batteries, supercapacitors, electrocatalysts, and energy storage because of supper metallic conductivity and surface activity, which was first reported in 2011 [19], [20], [21], [22], [23], [24]*.* Typically, MXenes were prepared through HF etching, ultrasonication, and exfoliation process to get ultrathin 2D nanosheets from three-dimension MAX. The present MAX is a layered hexagonal structure with a general formula of M_*n*+1_AX_*n*_ (*n* = 1, 2, and 3), where M refers to early transition metal, A represents group 13 and 14 elements, and X stands for carbon or nitrogen [25], [26], [27]. Owning to excellent features, such as hydrophilicity, biocompatibility, and high NIR absorption, MXenes, like Ti_3_C_2_, Ta_4_C_3_ [28], and Nb_2_C [29], have attracted considerable interest in biomedical application, especially tumor therapy.

MXene nanosheets have been used widely in tumor phototherapy because of high photothermal conversion efficiency, which also are the ideal drug delivery platform and less toxic to tissue [30], [31], [32], [33], [34]. Recently, some studies prepared MXene quantum dots (QDs) *via* new methods, such as Ti_2_N QDs [35] and Ti_3_C_2_ QDs [36], which exhibited high photothermal conversion efficiency and expressed only desired PTT effect *in vitro* and *in vivo* tests. Besides, most of the studies were aimed at achieving effective multifunctional tumor therapy through the modification of MXenes. Utilizing the magnetic resonance imaging capabilities of specific materials, the *in-situ* growth of superparamagnetic Fe_3_O_4_ nanoparticles on the surface of Ti_3_C_2_ was reported, which could obviously achieve efficient PTT due to the contrast-enhanced magnetic resonance imaging of tumors [37]. Moreover, some specific materials with the activity of Fenton reaction are applied to modify MXenes through the surface engineering, which have the ability to decompose H_2_O_2_ to generate highly toxic •OH, inducing tumor-cell apoptosis, achieving synergistic photothermal-enhanced nanocatalytic tumor therapy [38, 39]. Up to now, there are only a few reports on the improvements in the type II PDT efficiency of MXenes over a well-configured system through solving the hypoxia of tumor cells, yielding synergistic therapy of PDT/PTT under irradiation on NIR laser [40, 41].

**Figure 1:**
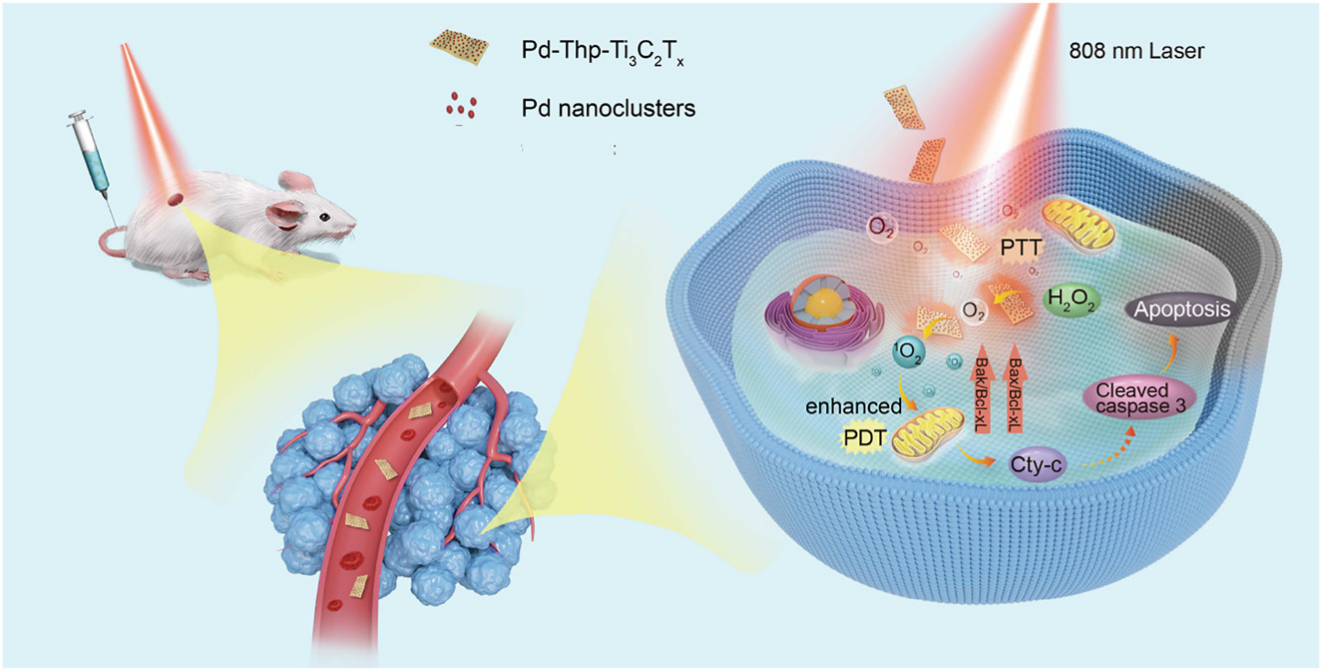
Schematic illustration of apoptosis mechanism mediated by synergistic PTT/PDT over Pd-Thp-Ti_3_C_2_T_*x*_ with enhanced nanocatalytic activity under NIR light.

In this study, Pd-Thp-Ti_3_C_2_T_*x*_ was synthesized with Ti_3_C_2_T_*x*_ nanosheets as carrier loading the photosensitizer, 5,10,15,20-Tetrakis(4-Aminophenyl)-21H,23H Porphyrin (Thp), and Pd nanoclusters. The nanocomposite exhibited some unique features: (1) Pd-Thp-Ti_3_C_2_T_*x*_ improved the stability and delivery efficiency of Thp; (2) compared with pure Ti_3_C_2_T_*x*_, Pd-Thp-Ti_3_C_2_T_*x*_ not only keep the high photothermal conversion efficiency, but also catalyzed excessive H_2_O_2_ of tumor cells to produce O_2_, resolving the hypoxic problems, hence improving the effect of PDT; (3) the nanocomposite effectually took advantage of the combined effect of PTT and enhanced PDT. Therefore, Pd-Thp-Ti_3_C_2_T_*x*_ produced effective therapeutic effect against hypoxic tumor. Pd-Thp-Ti_3_C_2_T_*x*_ nanocomposite could aggregate at tumor sites, as demonstrated by *in vivo* fluorescence imaging and photothermal imaging. In addition, *in vivo* toxicity evaluation tests indicated that nanocomposites had favorable biocompatibility and biosafety. Our study not only successfully achieves the synergistic therapy of PTT and enhanced PDT for solid tumor based on Ti_3_C_2_T_*x*_-based nanosheets (details are shown in Figure 1), but also provides guidance for the design of nanomaterials for cancer therapy.

## 2 Materials and methods (We have provided the assigned accreditation number of the laboratory (20200525002-GZ2021) for all the animal experiments.)

### 2.1 Preparation of Thp-Ti_3_C_2_T_*x*_ and Pd-Thp-Ti_3_C_2_T_*x*_

Typically, 300 μL of Thp (0.1 mg/mL) was added in the Ti_3_C_2_T_*x*_ (1 mg/mL) and was sonicated for 30 min, and then the solution was vibrated at 4 °C for 24 h. Finally, the sample was collected by centrifugation, and then the Thp-Ti_3_C_2_T_*x*_ was immersed into deionized water for 10 min sonication for later use. The Pd-Thp-Ti_3_C_2_T_*x*_ was also prepared *via* the same protocol.

### 2.2 Oxygen detection

For the detection of oxygen generation, 150 μL sample (1 mg/mL) was added into 3 mL of phosphate buffered saline (PBS, pH = 7) containing H_2_O_2_ (30 wt%) about 150 μL. The changes of dissolved oxygen concentration were tested by using JPB-607A within 20 min in the dark or under irradiation at 808 nm with 1.0 W/cm^2^.

### 2.3 Singlet oxygen generation

As the singlet oxygen (^1^O_2_) probe, 1,3-Diphenylisobenzofuran (DPBF) could react with ^1^O_2_ resulting the decrease for DPBF absorption at about 410 nm. Typically, 50 μL of DPBF (1 mg/mL in ethanol), 50 μL sample (1 mg/mL in deionized water) were added in the solution, in which the volume ratio of ethanol and deionized water is 1:1. The solution was irradiated under 808 nm laser with power density of 1.0 W/cm^2^ at different time point over 20 min, and then the spectra were recorded. For the study of effect of H_2_O_2_ in the experiments, another 3 μL of H_2_O_2_ was added into solution and collected the corresponding spectra.

### 2.4 Photothermal effect and photostability measurement

A certain number of nanocomposites dissolved in deionized water with different concentration were irradiated under 808 nm laser with certain power density. The temperature changes were monitored by FLIR infrared camera.

### 2.5 Cytotoxicity assay

The cytotoxicity analyses of different samples were performed *via* cell-counting kit 8 (CCK-8) assay. SW480, A549, MHCC97-L and CAKI cells were seeded in 96-well plates. After 24 h cultivation at 37 °C, the medium was replaced by medium including Pd-Thp-Ti_3_C_2_T_*x*_ with different concentration (0, 12.5, 25, 50, 100, 200 and 400 ppm) for 24 h at 37 °C, respectively. The mixture of CCK-8 and medium (1:10 of the volume ratio) were added wells staying for 3 h at 37 °C, and then the emitted fluorescence was measured by using a 96-well plate reader at 450 nm and the data were analyzed with Prism 7 software (GraphPad, Inc, La Jolla, CA, USA).

The photo-cytotoxicity on SW480 cells were also determined by CCK-8 assay. The previous processes are the same as above with different concentration of samples (0, 12.5, 25, 50, 100 ppm), after culture for 4 h, each well of experimental group was irradiated under 808 nm at 1.0 W/cm^2^ for 5 min, and the control group was placed aside but at dark environment. Then all 96-well plates were transferred to humidified incubator for 24 h. The medium was replaced by specific medium including 10% of CCK-8 for 3 h at 37 °C, and then the absorbance at 450 nm was recorded.

SW480 cells were inoculated in 96-well plates and cultured for 4 h in different medium containing nanomaterials (50 ppm). The cells were irradiated at 808 nm with 1.0 W/cm^2^ for 5 min and then cultured for 24 h. Finally, the living cells were stained with Calcein-AM and the dead cells were stained with propyl iodide (PI) for 30 min. Images were captured by Echo Laboratories Revolve FL.

### 2.6 *In vitro* PDT study

For ROS generation measurement, SW480 cells were cultured in medium with different sample (50 ppm) for 4 h at 37 °C with 5% CO_2_. The cells were irradiated under 808 nm laser at 1.0 W/cm^2^ for 5 min, and then washed with PBS for 2 times. The medium containing certain amount of 2,7-dichlorodihydro-fluorescein diacetate (DCFH-DA) were added staying for 20 min at 37 °C, and fluorescence images were collected by Echo Laboratories Revolve FL.

### 2.7 *In vivo* fluorescence imaging

For the fluorescence imaging of Pd-Thp-Ti_3_C_2_T_*x*_ *in vivo*, the mice model was established by intravenous injection with 100 µL Pd-Thp-Ti_3_C_2_T_*x*_-aminated sulfo-cyanine7 (Cy7) (2 mg/mL). For imaging, the 740 nm was used excitation and 760 nm as emission light was detected, and the *in vivo* images of mice injected at different time was recorded by a PerkinElmer IVIS Spectrum.

### 2.8 *In vivo* photothermal imaging and anti-tumor experiments

All female BALB/c mice were raised and purchased from Zhejiang Vital River Laboratory Animal Technology Co., Ltd. The tumor model was generated by injecting CT26 cells (2 × 10^6^ cells per mouse) in the back of each mouse. When the tumor volume reached about 50–100 mm^3^, the mice were randomly divided into 8 groups (*n* = 5): (1) PBS, (2) PBS + Laser, (3) Ti_3_C_2_T_*x*_, (4) Ti_3_C_2_T_*x*_ + Laser, (5) Thp-Ti_3_C_2_T_x_, (6) Thp-Ti_3_C_2_T_*x*_ + Laser, (7) Pd-Thp-Ti_3_C_2_T_*x*_, and (8) Pd-Thp-Ti_3_C_2_T_*x*_ + Laser. After intravenous injection for 4 h, the tumor was irradiated under 808 nm at 0.75 W/cm^2^ for 3 min, and the real-time infrared thermal images and tumor issue temperatures were recorded with an FLIR infrared camera. The reagents were intravenously injected about 100 μL (0.5 mg/mL) every other day for a total of 7 times over the treatment of 14 days. Meanwhile, the weight and tumor volume were measured and recorded. The relative volume was calculated as *V*/*V*_о_, where *V*_о_ and *V* represent the tumor volume on the first day and on the day of the measurement. After 14 days, all mice were euthanized, and the structure and states of the cells of tumor were analyzed *via* H&E staining and an anti-Ki-67 antibody. Meantime, the tumors were detected using the TUNEL method to analyze the fragments during apoptosis. And the major organs were subjected to H&E staining for histological analysis.

## 3 Results and discussion

### 3.1 Synthesis and characterization of the nanocomposites

**Figure 2:**
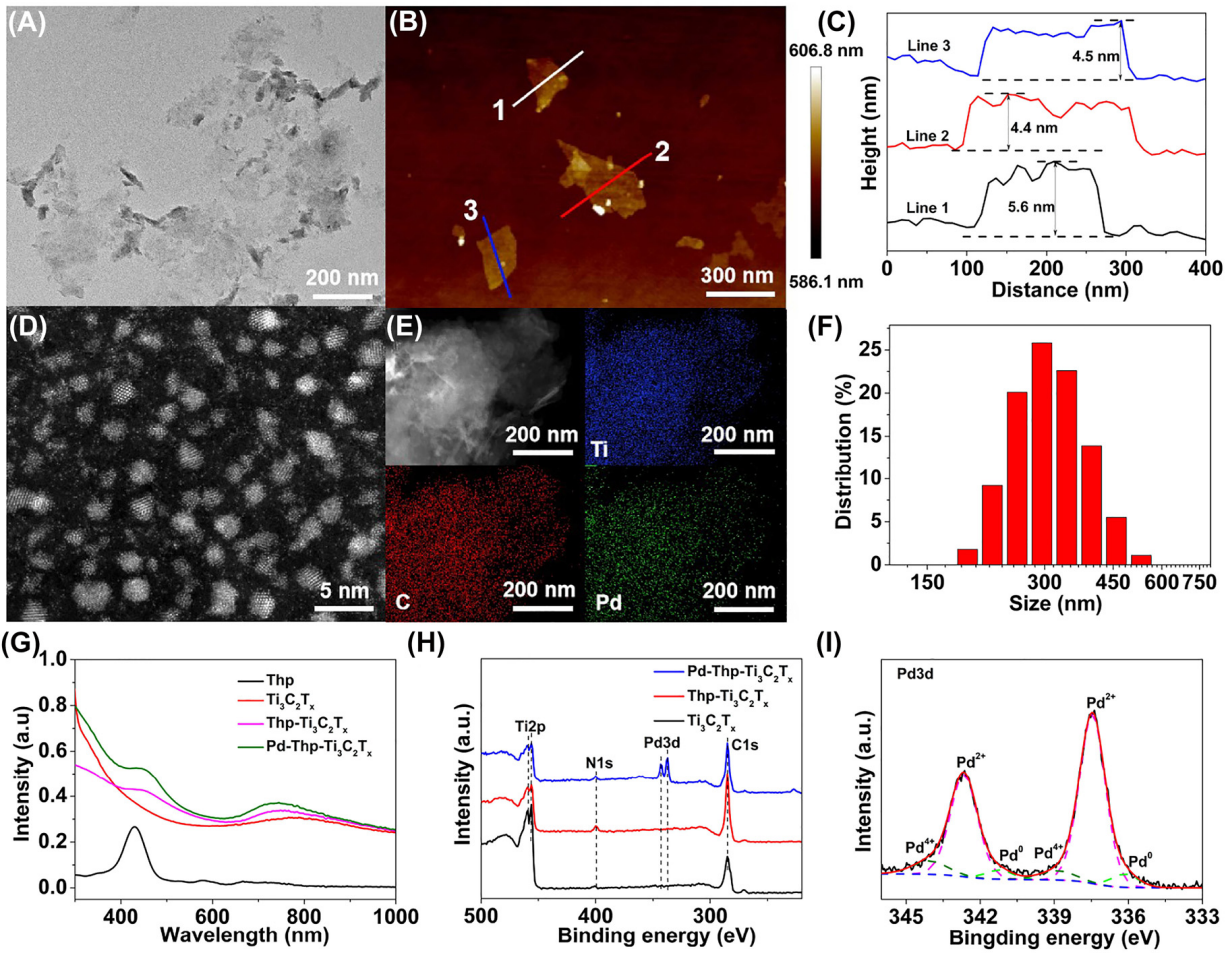
Geometric and electronic structure characterization of Pd-Thp-Ti_3_C_2_T_*x*_. (A) TEM images and (B) AFM image of Ti_3_C_2_T_*x*_ nanosheets. (C) AFM-measured thickness of Ti_3_C_2_T_*x*_ nanosheets. (D) and (E) HAADF-STEM images and corresponding EDS mapping images of Pd-Thp-Ti_3_C_2_T_*x*_. (F) Size distribution of Pd-Thp-Ti_3_C_2_T_*x*_ as determined by DLS. (G) UV–vis spectra of different nanocomposites. (H) XPS survey spectra of different nanocomposites. (I) High-resolution XPS spectra of Pd3d in Pd-Thp-Ti_3_C_2_T_*x*_.

As a MXene family member, Ti_3_C_2_T_*x*_ has been explored for tumor phototherapy because of its extraordinary photo-thermal conversion efficiency for PTT. However, its metallic conductivity hinders its application in PDT. In the study, a Pd-Thp-Ti_3_C_2_T_*x*_ nanocomposite was synthesized *via* a wet-chemistry approach to obtain highly efficient NIR PDT while preserving good PTT performance, releasing the synergistic effects of PTT and enhanced PDT for cancer. Pd was selected for its good performance in selectively catalyzing H_2_O_2_ into O_2_, which was further converted to ^1^O_2_ by Thp at 808 nm. The content of Pd measured about 1.7 wt% using inductively coupled plasma mass spectrometry (ICP-MS), and the loading amount of Thp of Thp-Ti_3_C_2_T_*x*_ and Pd-Thp-Ti_3_C_2_T_*x*_ was determined to be about 3 wt% according to the standard curve of free Thp (Figure S1). Transmission electron microscopy (TEM) images and DLS result showed that Ti_3_C_2_T_*x*_ had planer topology with lateral size of approximately 300 nm and thickness of about 5 nm, measured using atomic force microscopy (Figure 2A–C and Figure S2(A)). Figure 2D and E demonstrated that Pd nanoclusters were uniformly immobilized on Ti_3_C_2_T_*x*_ surface with a size of about 2 nm. TEM results displayed that Thp-Ti_3_C_2_T_*x*_ and Pd-Thp-Ti_3_C_2_T_*x*_ nanosheets had planer topology with lateral size of approximately 300 nm (Figure 2F and Figure S3). Moreover, Figure S2(B) showed that the lateral size of Pd-Thp-Ti_3_C_2_T_*x*_ was approximately 300 nm after preparation for 8 days, demonstrating the nanosheets possessed desired structural stability. The observation of the characteristic absorption peak of Thp for Thp-Ti_3_C_2_T_*x*_ and Pd-Thp-Ti_3_C_2_T_*x*_ indicated successful Thp loading onto nanocatalysts (Figure 2G). Moreover, the zeta potential of pure Ti_3_C_2_T_*x*_ nanosheets was −29.7 mV, which increased to −20.3 mV after Thp modification, and then decreased to −26.1 mV with the coating of Pd nanoclusters (Figure S4), showing that electrostatic interaction was probably the main factor for successful modification.

The chemical composition and valence states of elements in various composites were analyzed using X-ray photoelectron spectroscopy (XPS). As shown in Figure 2H, only the Pd3d peaks were observed in the XPS survey of Pd-Thp-Ti_3_C_2_T_*x*_, indicating the presence of Pd nanoparticles on the surface of Ti_3_C_2_T_*x*_ nanosheets. The fitted peaks of Pd3d indicated that Pd existed in the form of PdO_x_ (Figure 2I) [42], [43], [44]. The C1s spectra of pure Ti_3_C_2_T_*x*_ with four peaks located at 282.04, 284.79, 286.78, and 288.52 eV were assigned to C–Ti–T_*x*_, C=C, C–O, and C–F, respectively (Figure S5(A)) [45], [46], [47]. Compared with pure Ti_3_C_2_T_*x*_, the peak density of C–Ti–T_*x*_ of Thp-Ti_3_C_2_T_*x*_ and Pd-Thp-Ti_3_C_2_T_*x*_ decreased significantly as a result of the Thp and Pd cluster coated on the surface of Ti_3_C_2_T_*x*_ nanosheets (Figure S5(B) and (C)). The Ti2p XPS spectra of all samples fitted into eight peaks (Figure S5(D)–(F)), and the summary of deconvolution were listed in Table S1. Furthermore, compared with Ti_3_C_2_T_*x*_, the peak of the N1s of Thp-Ti_3_C_2_T_*x*_ and Pd-Thp-Ti_3_C_2_T_*x*_ shifted to high binding energy, demonstrating the electron transfer and interaction between Thp and Ti_3_C_2_T_*x*_ (Figure S6 and Table S2).

### 3.2 Optical properties of nanocomposites

**Figure 3:**
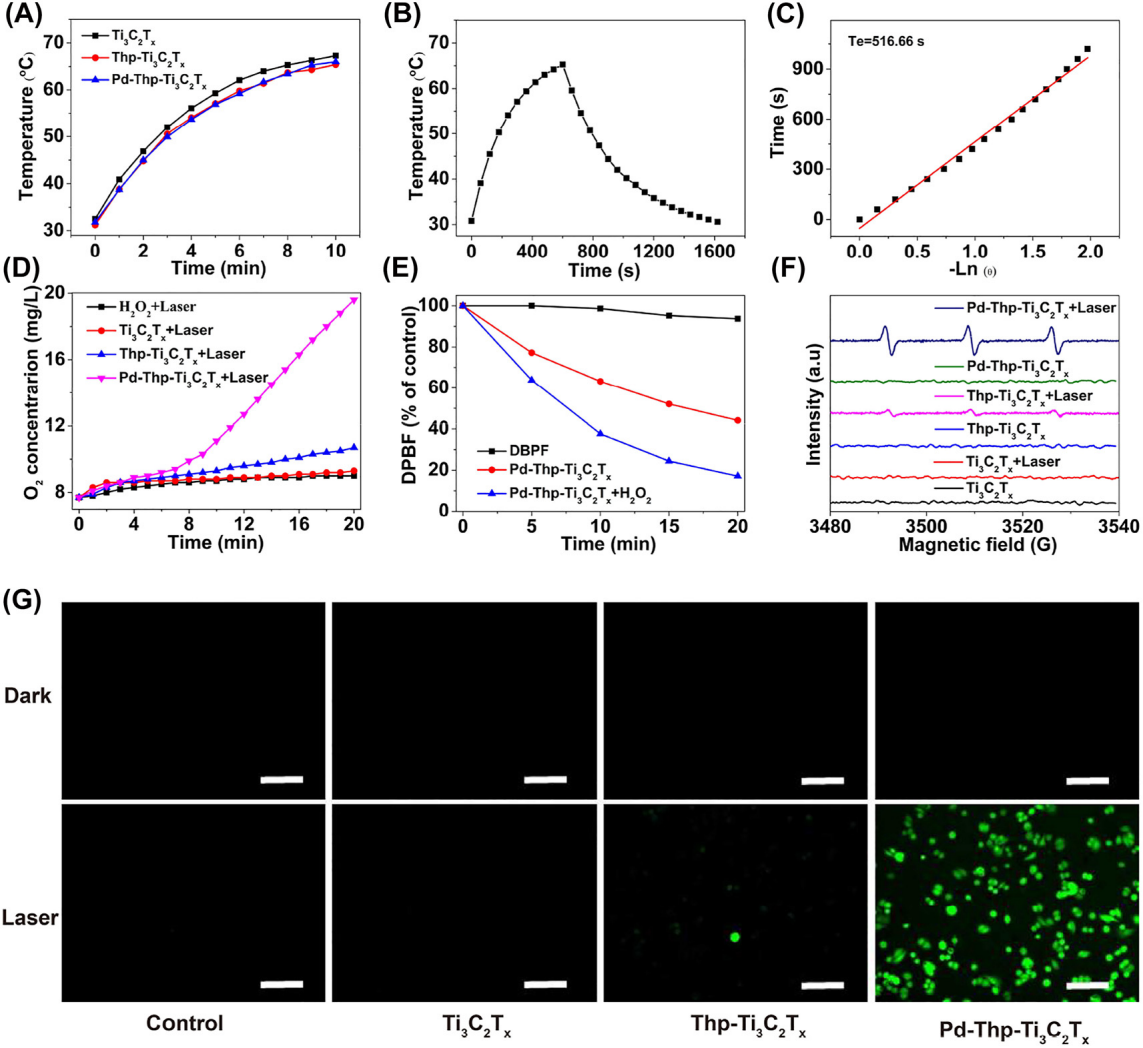
(A) Photothermal heating profiles of Ti_3_C_2_T_*x*_, Thp-Ti_3_C_2_T_*x*_, Pd-Thp-Ti_3_C_2_T_*x*_ (50 ppm). (B) Photothermal profile of Pd-Thp-Ti_3_C_2_T_*x*_ (50 ppm) under irradiation. (C) Heat-transferring time constant (*Tε*) obtained from the data of natural cooling period (R^2^ = 0.99). (D) O_2_ concentration of H_2_O_2_ solution after adding different nanocomposites under irradiation. (E) Decay curves of DPBF absorption at 410 nm in Pd-Thp-Ti_3_C_2_T_*x*_ with or without H_2_O_2_ after different time point of irradiation. (F) ^1^O_2_ generation for different nanocomposites characterized by ESR spectra in dark or under irradiation. (G) Intracellular ROS levels under irradiation were evaluated in SW480 cells by using DCFH-DA as the ROS probe (Scale bar: 100 μm). (Irradiation condition: 808 nm, 1.0 W/cm^2^, 5 min).

Recent reports indicate that Ti_3_C_2_T_*x*_ MXene shows high photothermal conversion property with the photothermal conversion efficiency (*η*) up to 102.7% [48]. The photothermal effect of nanocomposites mainly depended on two parameters containing *η* and the extinction coefficient (*ε*). The UV–vis-NIR profile of Pd-Thp-Ti_3_C_2_T_*x*_ showed the obvious absorption at NIR-I biowindow around 808 nm (Figure S7(A) and (B)), and the *ε* of Pd-Thp-Ti_3_C_2_T_*x*_ was calculated to be 6.38 L/g/cm, which was higher than that of state-of-art nanoagents, like graphene oxide (3.6 L/g/cm) [49]. As shown in Figure 3A, the temperatures of Ti_3_C_2_T_*x*_, Thp-Ti_3_C_2_T_*x*_, and Pd-Thp-Ti_3_C_2_T_*x*,_ were up to about 66.0 °C under the same irradiation condition, indicating that the modification had no significant influence on the photothermal property of Ti_3_C_2_T_*x*_. Meanwhile, the photothermal property of Pd-Thp-Ti_3_C_2_T_*x*_ at different concentration and laser power density was studied systematically. Figure S7(C) showed that the temperature of Pd-Thp-Ti_3_C_2_T_*x*_ with 12.5 ppm at 808 nm for 10 min reached 48.9 °C and was elevated to 80.7 °C at 200 ppm. In contrast, the temperature of deionized water after irradiation of the same condition underwent no obvious changes. These results indicated Pd-Thp-Ti_3_C_2_T_*x*_ had concentration-dependent photothermal features and could serve as the potential photothermal-conversion nanoagent. As expected, Pd-Thp-Ti_3_C_2_T_*x*_ also exhibited power density-dependent photothermal feature (Figure S7(D)), with the solution temperature reaching up to 41.0 °C at 0.2 W/cm^2^ and to 87.8 °C at 2.0 W/cm^2^. In particular, the *η* of Pd-Thp-Ti_3_C_2_T_*x*_ was measured to be almost 62.1%, higher than that of most nanoagents, such as BP QDs (28.4%), TiN NPs (48.0%), and MoSe_2_ nanodots (Figure 3B and C) [50], [51], [52]. Notably, the Pd-Thp-Ti_3_C_2_T_*x*_ showed stable absorbance at 808 nm and photothermal performance (Figures S8 and S9). Therefore, the prepared Pd-Thp-Ti_3_C_2_T_*x*_ nanosheets revealed perfect photothermal conversion effect, which guaranteed good photothermal-hyperthermia performance on the ablation of tumors.

For the nanocomposite, the capability for decomposing H_2_O_2_ into O_2_ and H_2_O to release hypoxia was investigated quantitatively. As expected, a large number of bubbles were rapidly generated after the addition of Pd-Thp-Ti_3_C_2_T_*x*_ into the H_2_O_2_ solution, but there was almost no generation of O_2_ bubbles in the presence of Ti_3_C_2_T_*x*_ and Thp-Ti_3_C_2_T_*x*_, H_2_O and H_2_O_2_ solution only after the same time (Figure S10). In addition, an oxygen probe (JPB-607A) was used to measure the O_2_ content of the solution. As displayed in Figure S11, the laser irradiation could decompose a small amount of H_2_O_2_ to generate O_2_. In the absence of irradiation, Pd-Thp-Ti_3_C_2_T_*x*_ showed better catalytic activity for O_2_ production than other nanocomposites (Figure S12). After 808 nm irradiation, Pd-Thp-Ti_3_C_2_T_*x*_ could catalyze H_2_O_2_ to produce more O_2_ (Figure 3D), but the increased O_2_ content in Ti_3_C_2_T_*x*_ and Thp-Ti_3_C_2_T_*x*_ was nearly similar to the amount of O_2_ produced by H_2_O_2_ decomposition only under irradiation for 20 min. The results demonstrated that Pd-Thp-Ti_3_C_2_T_*x*_ could effectively decompose H_2_O_2_ to generate O_2_, indicating the potential performance of PDT for hypoxic tumors.

ROS accounts for the efficacy of PDT to kill tumor cells, which can cause irreversible damage to important organelles or the DNA, eventually leading to cell apoptosis and necrosis. Thus, 1,3-Diphenylisobenzofuran (DPBF) was used as ^1^O_2_ detector to investigate the ^1^O_2_-production capacity of these nanocomposites, which can react with ^1^O_2_ to reduce the absorption peak density to about 410 nm. First, the absorption peak of DPBF solution without the addition of any nanocomposite did not decrease significantly under laser irradiation, showing that negligible ^1^O_2_ was generated in the process (Figure S13). In the presence of Ti_3_C_2_T_*x*_, the results showed that there was no evidence of ^1^O_2_ generation (Figure S14(A)). To our satisfactory, in the presence of Thp-Ti_3_C_2_T_*x*_ and Pd-Thp-Ti_3_C_2_T_*x*_, the absorption peak of DPBF at 410 nm decreased gradually with the increase in irradiation time (808 nm, 1.0 W/cm^2^) (Figure S14(D) and (G)). Compared with Thp-Ti_3_C_2_T_*x*_, Pd-Thp-Ti_3_C_2_T_*x*_ was more efficient in the production of ^1^O_2_ with the same content of photosensitizer, Thp, showing that Pd modification improved the generation of ^1^O2. Meanwhile, the influence of H_2_O_2_ was investigated. Following the addition of H_2_O_2_, the ^1^O_2_ generation efficiency of Thp-Ti_3_C_2_T_*x*_ and Pd-Thp-Ti_3_C_2_T_*x*_ improved significantly (Figure 3E, Figure S14(E), (F) and (H)). In the first 5 min, the formation of ^1^O_2_ of Pd-Thp-Ti_3_C_2_T_*x*_ was significantly more than that of Thp-Ti_3_C_2_T_*x*_, indicating that Pd-Thp-Ti_3_C_2_T_*x*_ served as an oxygen self-supply nano-factory through the decomposition of H_2_O_2_ into O_2_ over Pd nanoclusters, which was continuously transformed into ^1^O_2_. In contrast, the absorption peaks for Ti_3_C_2_T_*x*_ were almost unchanged in the presence of H_2_O_2_ (Figure S14(B) and (C)), meaning that the Ti_3_C_2_T_*x*_ used in this research could not effectively catalyze H_2_O_2_ to generate O_2_ at the reaction condition. The remaining TEMP spin-trapping ESR also confirmed that the most ^1^O_2_ was formed with Pd-Thp-Ti_3_C_2_T_*x*_ under 808 nm irradiation with 1.0 W/cm^2^ (Figure 3F).

In addition, as an ROS detection probe, 2,7-dichlorodihydro-fluorescein diacetate (DCFH-DA) was used to research the intercellular ROS generation in human colon cancer cells, SW480, which could be oxidized by ROS to form fluorescent DCF and further emit bright green fluorescence in cells. In Figure 3G, there was almost no green fluorescence in the group without laser irradiation, treated with Ti_3_C_2_T_*x*_ and Thp-Ti_3_C_2_T_*x*_ under irradiation (808 nm, 1.0 W/cm^2^, 5 min). Nevertheless, bright green fluorescence was observed in SW480 cells treated with Pd-Thp-Ti_3_C_2_T_*x*_ under the same irradiation conditions. The results showed that Pd-Thp-Ti_3_C_2_T_*x*_ produced more ROS under NIR irradiation resulting in the efficient decomposition of H_2_O_2_ into O_2_, which was potent for cancer PDT.

### 3.3 *In vitro* anticancer performance and cellular uptake of nanocomposites

**Figure 4:**
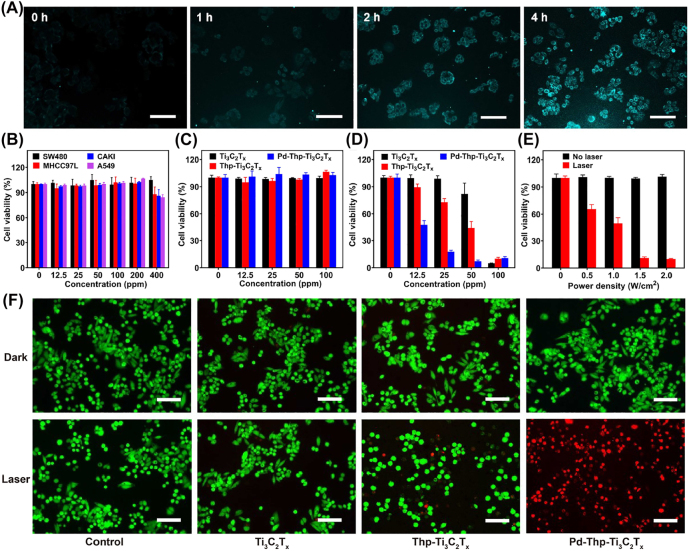
*In vitro* evaluation of the cytotoxicity for cancer cells under NIR irradiation. (A) SW480 cells incubated with Pd-Thp-Ti_3_C_2_T_*x*_-Cy5.5 (50 ppm) for different time points. (B) Cell viabilities of SW480, CAKI, MHCC97-L and A549 cells incubated with Pd-Thp-Ti_3_C_2_T_*x*_ with different concentration (0, 12.5, 25, 50, 100, 200 and 400 ppm). Cell viabilities of SW480 cells incubated with different composites at different concentration under (C) no laser and (D) laser (irradiation conditions: 808 nm, 1.0 W/cm^2^ and 5 min). (E) Cell viabilities of SW480 cells incubated with Pd-Thp-Ti_3_C_2_T_*x*_ under 808 nm for 5 min with different power density. (F) The microscopy images of SW480 cells stained with AM/PI after different treatments (irradiation conditions: 808 nm, 1.0 W/cm^2^, 5 min; Scale bar: 100 μm).

To study the cellular uptake of nanocomposites, cyanine 5.5 (Cy5.5) was used as fluorescence probe to synthesize Pd-Thp-Ti_3_C_2_T_*x*_-Cy5.5. As shown in Figure 4A, the fluorescence intensity was gradually enhanced within 4 h of incubation, revealing that Pd-Thp-Ti_3_C_2_T_*x*_ could be efficiently endocytosed into SW480 cells. In addition, cell-counting kit 8 (CCK-8) assay was used to measure the cytotoxicity of nanocomposites. Following the cultivation of Pd-Thp-Ti_3_C_2_T_*x*_ at different concentrations (0, 12.5, 25, 50, 100, 200, and 400 ppm) for 24 h, negligible toxicity to SW480, CAKI, MHCC97-L, and A549 cells was found (Figure 4B), and the cell abilities were still above 85% even at a high concentration of 400 ppm, indicating the excellent cytocompatibility of Pd-Thp-Ti_3_C_2_T_*x*_. Subsequently, the phototoxicity of nanocomposites on SW480 cells was investigated, and no significant decrease in cell viability was found in all the groups without laser irradiation (Figure 4C). In contrast, after irradiation under 808 nm laser at 1.0 W/cm^2^ for 5 min, almost 60% of the cells treated with Pd-Thp-Ti_3_C_2_T_*x*_ were killed, which was much higher than those treated with Thp-Ti_3_C_2_T_*x*_ (12%) and Ti_3_C_2_T_*x*_ (0%) with the same concentration of 12.5 ppm, and the phototherapy efficacy of Pd-Thp-Ti_3_C_2_T_*x*_ was positively correlated with concentration and laser intensity (Figure 4D and E). Notably, with the same concentration of Thp, Thp-Ti_3_C_2_T_*x*_, and Pd-Thp-Ti_3_C_2_T_*x*_ showed better performance in killing SW480 cells, which might be due to Thp loading on Ti_3_C_2_T_*x*_ improving its stability and delivery efficiency (Figure S15). Meanwhile, SW480 cells incubated with nanocomposites were exposed to 808 nm laser at 1.0 W/cm^2^ for 5 min and were then co-stained with Calcein-AM (green fluorescence) and propidium iodide (PI: red fluorescence) which could label the living and dead cells. As shown in Figure 4F, strong green fluorescence was detected in all the groups in the absence of 808 nm irradiation, demonstrating that the viability of cells was not obviously affected. However, after irradiation, no red fluorescence was detected in the control group, and a faint red fluorescence signal was displayed in cells incubated with Ti_3_C_2_T_*x*_ and Thp-Ti_3_C_2_T_*x*_. But SW480 cells incubated with Pd-Thp-Ti_3_C_2_T_*x*_ emitted stronger red fluorescence, illustrating the efficient phototherapy performance of Pd-Thp-Ti_3_C_2_T_*x*_ and revealing that Pd-Thp-Ti_3_C_2_T_*x*_ had negligible cytotoxicity and high phototoxicity.

**Figure 5:**
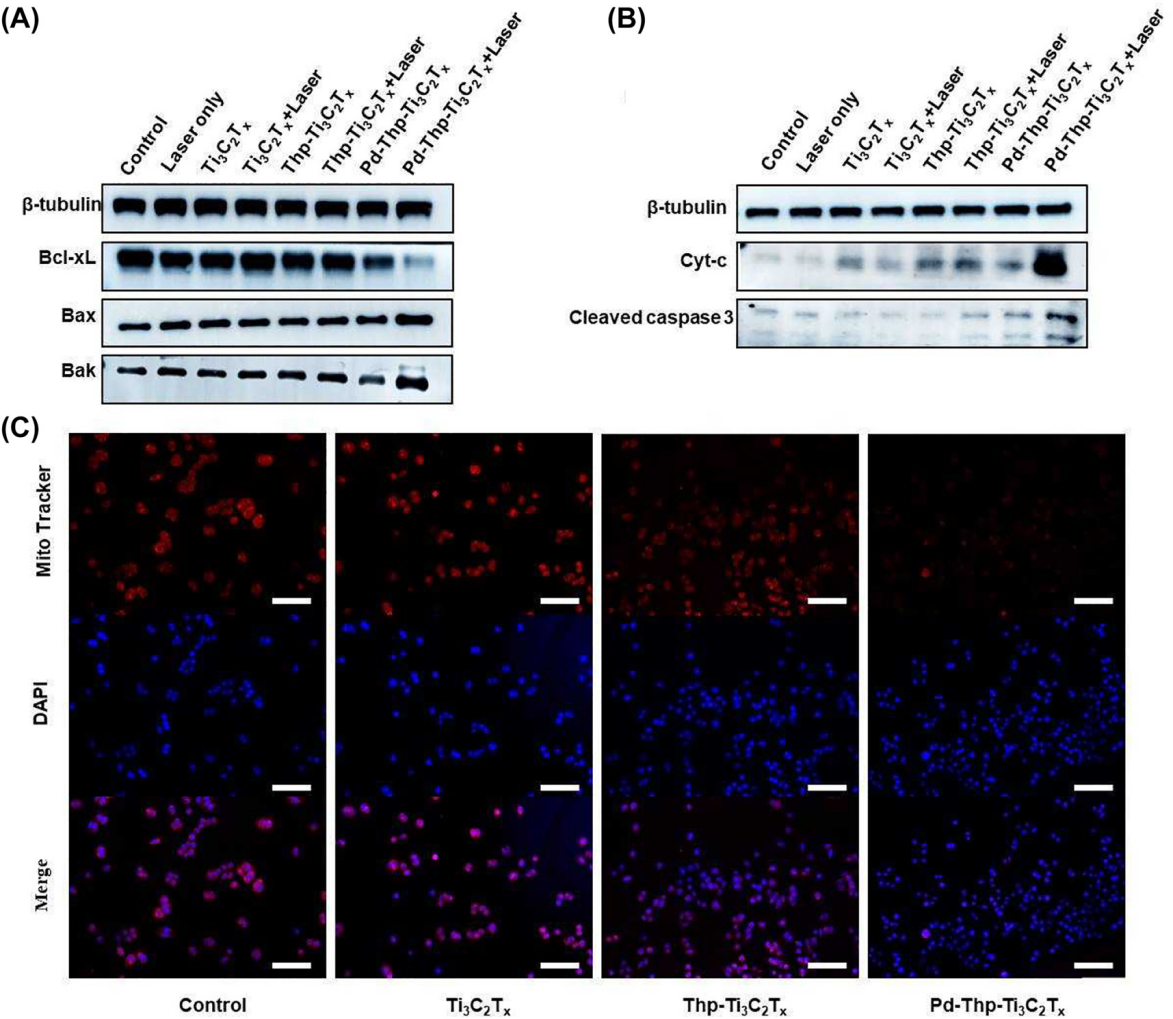
(A) Expression of Bcl-2 family protein Bax, Bak and Bcl-xL by western blotting using SW480 cells. (B) Expression of Cyt-c and cleaved caspase 3 by western blotting using SW480 cells. (C) Cells treated with different composites were irradiated under 808 nm and stained with MitoTracker Red CMXRos. (Treatment condition: 12.5 ppm, 808 nm, 1.0 W/cm^2^, 5 min; scale bar: 100 μm).

The cell apoptosis pathway reported for most nanomaterials follows the mitochondrial control of apoptosis [53]. To further investigate the mechanism of Pd-Thp-Ti_3_C_2_T_*x*_-induced SW480 cell apoptosis, some parameters were examined, including Bak, Bax, Bcl-xL, cytochrome c (Cyt-c), and cleaved caspase 3. Generally, the Bcl-2 family proteins are classified into two kinds according to structure and function, namely anti-apoptosis protein (Bcl-2, Bcl-xL, Bcl-w, etc.) and pro-apoptosis protein (Bax, Bak, Bid, Bim, etc.). As shown in Figure 5A, the expression of Bax and Bak increased in tumor cells treated with Pd-Thp-Ti_3_C_2_T_*x*_ under 808 nm irradiation, while Bcl-xL was downregulated significantly, and the others had almost no change. Meanwhile, the upregulation of Bax/Bcl-xL and Bak/Bcl-xL revealed that Pd-Thp-Ti_3_C_2_T_*x*_-induced PDT/PTT had apoptosis-promoting effect, which could make mitochondria dysfunctional, accelerating the release of Cyt-c from mitochondria into cytoplasm. As expected, Cyt-c in the cytoplasm was found to increase significantly (Figure 5B), and this could lead to the activation of caspases in the apoptosome as a pivotal effector of apoptosis. Caspase 3 is the main effector caspase that is involved in apoptosis, which could be active to generate cleaved caspase 3, leading to the cleavage of cellar substrates and apoptosis. Just as shown in Figure 5B, the expression of cleaved caspase 3 increased in cells cultured with Pd-Thp-Ti_3_C_2_T_*x*_. Meanwhile, MitoTracker Red CMXRos was used to examine changes in the mitochondrial membrane, which is an indicator of mitochondrial damage. As shown in Figure S16, SW480 cells cultured with different samples without laser irradiation emitted obvious red fluorescence. It is worth noting that, after 808 nm laser irradiation, the intensity of red fluorescence decreased significantly, especially for Pd-Thp-Ti_3_C_2_T_*x*_ (Figure 5C), which manifests the dysfunction of the mitochondrial membrane. Altogether, the results showed that the PDT and PTT induced by Pd-Thp-Ti_3_C_2_T_*x*_ had obvious pro-apoptosis effects for SW480 cells through the mitochondria control of apoptosis.

### 3.4 *In vivo* biosafety of nanocomposites

**Figure 6:**
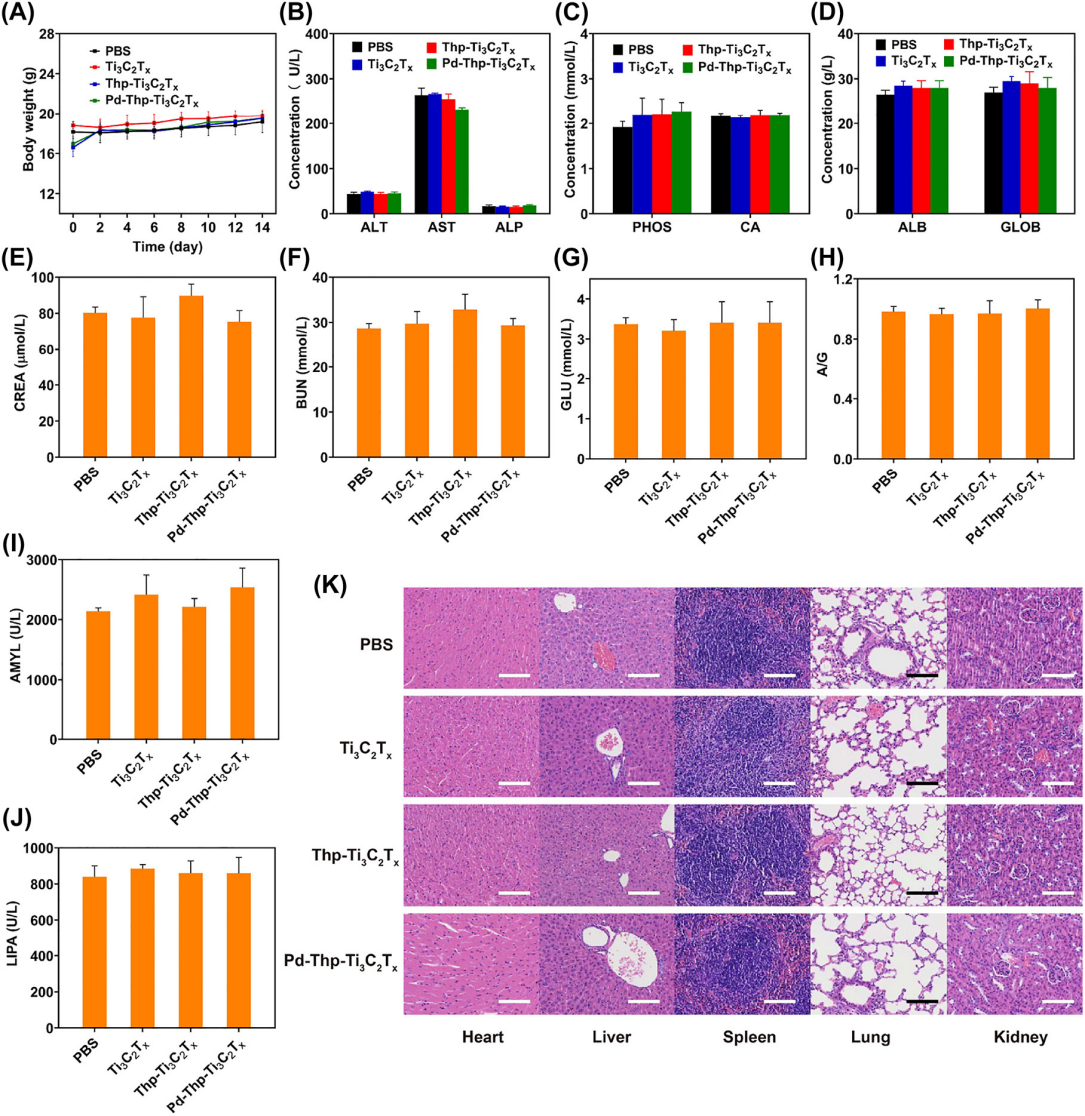
*In vivo* biosafety evaluation of various nanocomposites. (A) Time-related body weight of BALB/c mice after the intravenous administration with different nanocomposites. (B–J) Blood biochemical analysis of BALB/c mice after the intravenous administration with different composites at 14th day. (K) H&E-stained tissue sections of major organs from BALB/c mice in biosafety experiments (Scale bar: 100 µm).

Prior to the application of the nanocomposites in solid tumor, the biosafety analyses were performed through one-time intravenous injection of different nanocomposites at 20 mg/kg into healthy female BALB/c mice. There were no obvious changes in the body weight of the mice in all the groups within 14 days (Figure 6A), meaning that Ti_3_C_2_T_*x*_, Thp-Ti_3_C_2_T_*x*_, and Pd-Thp-Ti_3_C_2_T_*x*_ had neglectable influence on the growth of the mice. Moreover, standard hematology, including some blood biochemical parameters, was tested to investigate the potential impact of nanocomposites. As shown in Figure 6B, E and F, the aspartate aminotransferase (AST), alkaline phosphatase (ALP), alanine aminotransferase (ALT), urea nitrogen (BUN), and creatinine (CREA) were not different from those of the PBS group, which meant that the nanocomposites had negligible adverse effects on the liver and kidneys. Moreover, albumin (ALB), globulin (GLOB), ALB/GLOB (A/G), lipase (LIPA), amylopsin (AMYL), blood glucose (GLU), calcium ion (CA), and phosphonium ion (PHOS) levels also had no significant changes compared to the PBS group (Figure 6C, D and G–J). These results showed that the nanocomposites had no remarkable toxicity effects on the mice at the given dose. In addition, as shown in Figure 6K, compared with healthy mice of the same age, the results of hematoxylin and eosin (H&E) staining of major organs, including heart, liver, spleen, lung, and kidney, shows that there are no obvious damages of cell or tissue. These data unambiguously demonstrates that Ti_3_C_2_T_*x*_-based nanomaterials are biocompatible, with potential for application in cancer phototherapy.

### 3.5 *In vivo* fluorescence imaging, photothermal imaging, and anticancer activity of nanocomposites

**Figure 7:**
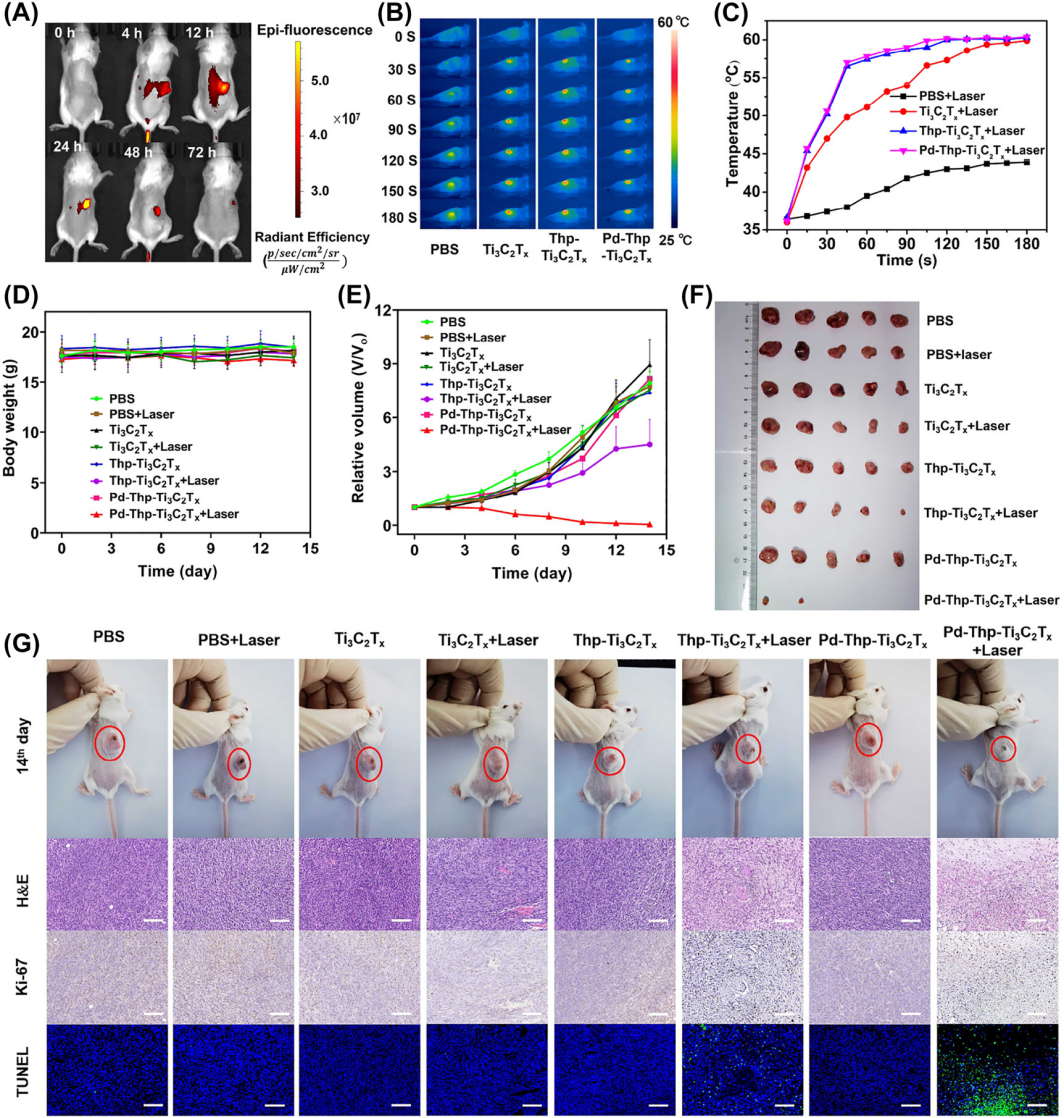
*In vivo* synergistic therapy of PTT and enhanced PDT of Pd-Thp-Ti_3_C_2_T_*x*_ for anticancer therapy. (A) *In vivo* fluorescence imaging of CT26 tumor model after intravenous injection of Thp-Ti_3_C_2_T_*x*_-Cy7 at different time. (B) IR photothermal images of CT26-tumor-bearing mice in different groups, including PBS + Laser, Ti_3_C_2_T_*x*_ + Laser, Thp-Ti_3_C_2_T_*x*_ + Laser and Pd-Thp-Ti_3_C_2_T_*x*_ + Laser, and (C) corresponding temperature curve of the tumor sites. (D) Time-depending body weight and (E) time-related relative tumor volume of CT26-tumor-bearing mice and followed with or without 808 nm irradiation (irradiation condition: 808 nm, 0.75 W/cm^2^, 5 min). (F) Morphology of the tumors removed from the BALB/c mice from all group at 14th day of this work. (G) Digital images of CT26-tumor-bearing mice and its tumor region at 14th day, and H&E, Ki-67 and TUNEL staining on the dissected tumor tissues (Scale bar: 100 µm).

In order to reveal its potential in clinical cancer therapy, the distribution of nanocomposites in mice body after intravenous injection was first investigated. Pd-Thp-Ti_3_C_2_T_*x*_-Cy7 was prepared using aminated sulfo-cyanine7 (Cy7) as fluorescent labeled molecule. As Figure 7A displayed, the fluorescent intensity attained the highest value at 24 h post-injection, and then decreased gradually, which was possibly due to the metabolism of the mice body and the biodegradation of the composites. Besides, we verified the *in vivo* tumor accumulation of the Pd-Thp-Ti_3_C_2_T_*x*_-Cy7 and their *in vivo* targeting ability using an *ex vivo* investigation of the major organs 72 h post-injection. It was found that there is a certain degree of accumulation in the liver and kidney tissues (Figure S17), which is an issue encountered by many other nanomedicines. The biodegradation property could guarantee the biosafety of the nanocomposite. These results proved that nanomaterials could aggregate at the tumor site, ensuring subsequent effective *in vivo* phototherapy.

Based on the above results, the *in vivo* anti-tumor effects of Pd-Thp-Ti_3_C_2_T_*x*_ were investigated in more detail. To this end, female BALB/c mice with CT26 tumor model were randomly divided into 8 groups (*n* = 5): (1) PBS, (2) PBS + Laser, (3) Ti_3_C_2_T_*x*_, (4) Ti_3_C_2_T_*x*_ + Laser, (5) Thp-Ti_3_C_2_T_*x*_, (6) Thp-Ti_3_C_2_T_*x*_ + Laser, (7) Pd-Thp-Ti_3_C_2_T_*x*_, and (8) Pd-Thp-Ti_3_C_2_T_*x*_ + Laser. Over the treatment period of 14 days, the reagents were intravenously injected every other day for a total of 7 times. After 4 h *via* tail intravenous injection every time, the tumor was exposed to 808 nm with 0.75 W/cm^2^ for 3 min, and the real-time infrared thermal images were recorded (Figure 7B). Photothermal imaging is helpful to observe the real-time effect of tumor treatment more intuitively. The tumor surface temperature of groups (4), (6), and (8) increased to 60 °C (Figure 7C), which was high enough to ablate the tumor cells. However, for the control group without injection of any nanocomposites under the same irradiation condition, the tumor temperature increased from 37 °C to 43 °C, while the slight increase in temperature could not make the death of tumor cells. The results revealed that the Pd-Thp-Ti_3_C_2_T_*x*_ showed obvious photothermal effect and could be used as photothermal imaging. During the therapeutic period, the body weight of the mice in all the groups showed no obvious changes (Figure 7D), indicating that the dosage of nanocomposites had no significant effect on healthy growth of the mice. For the tumor volume, the groups without irradiation, PBS + Laser and Ti_3_C_2_T_*x*_ + Laser, showed fast growth (Figure 7E), revealing that the PTT of Ti_3_C_2_T_*x*_ + Laser alone could not restrain the tumor growth. In contrast, the tumor suppression rate of Pd-Thp-Ti_3_C_2_T_*x*_ + Laser group could be up to 100%, however, the value was only 50% for Thp-Ti_3_C_2_T_*x*_ + Laser group, which was due to the fact that Pd-Thp-Ti_3_C_2_T_*x*_ could decompose excess H_2_O_2_ of tumor cells into O_2_, improving the PDT efficiency, leading to better synergistic therapy of enhanced PDT and PTT. These results are also supported by the representative tumor images and digital images of all groups on the 14th day (Figure 7F and S18(A)), revealing the outstanding catalytic activity and photothermal conversion of modified Ti_3_C_2_T_*x*_, and achieving desirable effect of enhanced PDT and high-efficiency PTT.

Once the phototherapy treatment was conducted, the tumors were dissected and the pathological evaluation and therapeutic efficacy was performed using H&E, antigen Ki-67 expression, and TUNEL staining. Comparatively, large tumor cells damage and decrease in cell proliferation were recorded for the Thp-Ti_3_C_2_T_*x*_ + Laser and Pd-Thp-Ti_3_C_2_T_*x*_ + Laser groups (Figure 7G). In addition, the Pd-Thp-Ti_3_C_2_T_*x*_ + Laser group had more severe cell damage. Meanwhile, the Pd-Thp-Ti_3_C_2_T_*x*_ + Laser group indicated a large amount of cell apoptosis with the nuclei being stained with remarkable cyan. These results aptly confirmed that Pd-Thp-Ti_3_C_2_T_*x*_ could catalyze H_2_O_2_ into O_2_, which relieved the hypoxic environment of the solid tumor, in turn improving the effect of PDT, resulting in efficient apoptosis of tumor cells combined with PTT. For further application of this nanocomposite *in vivo*, the potential toxicity needs to be analyzed. The H&E staining of major organs showed no obvious toxic side effects (Figure S18(B)), suggesting that the Pd-Thp-Ti_3_C_2_T_*x*_ had no noticeable *in vivo* potential toxicity.

## 4 Conclusions

In summary, a single NIR-responsive oxygen self-supply Pd-Thp-Ti_3_C_2_T_*x*_ 2D nanocomposite was developed using a rationally designed method, which took advantage of the unique photothermal conversion efficiency of Ti_3_C_2_T_*x*_ and relived the hypoxic microenvironment of solid tumor *via* catalysis to enhance the PDT effect of photosensitizer. Compared with most traditional 2D Ti_3_C_2_T_*x*_ with only PTT effect, this nanocomposite is endowed with admirable catalytic performance and broadens the biomedical applications of such excellent optical materials. Through the modification of Pd nanoclusters, Pd-Thp-Ti_3_C_2_T_*x*_ had catalytic activity of decomposing excess H_2_O_2_ in tumor cells to produce O_2_, releasing *in situ* oxygen supply for the photosensitizer action. Meanwhile, photosensitizer Thp loaded on the surface of MXene made them more easily prone to endocytosis by the cell to realize enhanced NIR PDT. The Pd-Thp-Ti_3_C_2_T_*x*_ nanoplatform was successfully employed in tumor *in vivo* PT imaging. *In vitro* and *in vivo* experiments indicated that the Pd-Thp-Ti_3_C_2_T_*x*_ had high biocompatibility and ignorable toxicity and achieved the combined effect of PTT and enhanced PDT for cancer therapy. Hypoxia restricts different disease therapies, and thus, the oxygen self-supply metal-dopant/MXene nanocomposite and it not only improves the PDT performance by loading photosensitizes, but also shows the potential application of Ti_3_C_2_T_*x*_-based nanoplatform in more cancer therapy *via* surface engineering (e.g., enhance immune therapy by replacing the photosensitizer with immune checkpoint inhibitors).

## Supplementary Material

Supplementary Material Details
