# Supplementary material for: Three birds with one stone: oxygen self-supply engineering palladium nanocluster/titanium carbide hybrid for single-NIR laser-triggered synergistic photodynamic-photothermal therapy
Source: Nanophotonics. 2022 Aug 5;11(22):5061–75. doi: 10.1515/nanoph-2022-0268 (PMC11501429; doi:10.1515/nanoph-2022-0268)
Supplement: Supplementary file 1 — Supplementary Material Details [file j_nanoph-2022-0268_suppl.docx]

**Supporting Information**

Shanshan Dang, Yanmei Mo, Junqing Zeng , Yunjie Xu, Zhongjian Xie, Han Zhang*, Bin Zhang*, Guohui Nie*

**Three birds with one stone: oxygen self-supply engineering palladium nanocluser/titanium carbide hybrid for single-NIR laser-triggered synergistic photodynamic-photothermal therapy**

Dr. S. Dang, Ms. Y. Mo, Dr. Y. Xu, Prof. Z. Xie, Prof. H. Zhang, Prof. B. Zhang, Prof. G. Nie

Shenzhen Key Laboratory of Nanozymes and Translational Cancer Research, Institute of Translational Medicine Department of Otolaryngology Shenzhen Second People’s Hospital, the First Affiliated Hospital of Shenzhen University, Health Science Center, Institute of Microscale Optoelectronics Shenzhen 518035, China

Mr J. Zeng

Graduate Collaborative Training Base of Shenzhen Second’ People’s Hospital, Hengyang Medical School, University of South China, Hengyang, Hunan, 421001, China

***Corresponding Authors**: Han Zhang ([hzhang@szu.edu.cn](mailto:hzhang@szu.edu.cn)), Bin Zhang ([wenwubin09@163.com](mailto:wenwubin09@163.com)), Guohui Nie ([nieguohui@email.szu.edu.cn](mailto:nieguohui@email.szu.edu.cn))

**Experimental details**

**Materials**

Ti_3_C_2_T_x_ (solution), 5,10,15,20-Tetrakis (4-Aminophenyl)-21H,23H Porphyrin (Thp), 3-Mercaptopropionic acid (MPA), and Palladium nitrate solution (Pd 18.09 wt% in nitric acid) were purchased from CAS-mart (China). All chemicals were purchased from commercial supplies and used without further treatment.

**Preparation of monodispersed Pd catalyst**

0.25 mL of Pd(NO_3_)_2_ aqueous solution, 2 ml of MPA solution were added in 2.35 mL of deionized water under magnetic stirring for 5 min, and then 0.3 mL of NaOH (1M) were added drop by drop to the mixture for another 5 min. Afterwards, a certain amount of NaBH_4_ solution was dissolved in the solution. After it was kept under stirring at room temperature for 2 h, the dark yellow solution was transferred to the dialysis bag and performed in deionized water to remove the reductant ions for 6 h, and then the monodispersed Pd catalyst in water with negligible NaOH, MPA and NaBH_4_ was obtained and stored at ­-20 ^o^C for further use.

**Characterization**

X-Ray photoelectron spectrum (XPS) was recorded by a Thermo Scientific Escalab 250Xi. The morphology of samples and energy X-ray dispersive spectroscopic (EDX) were performed by a Hitachi High-Tech's scanning electron microscopes SU3800. The transmission electron microscope (TEM) images were obtained on HITACHI Transmission Electron Microscope (HT7700), and the nanostructures of catalysts were performed by High-resolution Transmission electron microscope (HRTEM) images and element Mapping were performed by a field emission transmission electron microscope (JEOL EM F200). The Pd loading of Pd-Thp-Ti_3_C_2_T_x_ was determined by inductively coupled plasma (ICP) analysis on a Thermo Scientific Escalab 250Xi. UV-vis-NIR absorption spectra were recorded by a Evolution220 ThermoFisher. Size and Zeta potential characterization were measured on Zetaszier Nano-ZS90. The thickness of nanosheets was measured via atomic force microscope (AFM) by Dimension icon (Bruker). TEMP electron spin-resonance spectroscopy (ESR) was performed by Bruker E500.

**Cell culture**

SW480, A549, MHCC97L and CAKI cells were cultured in DEME supplemented with 10% fetal bovine serum and 1% penicillin-streptomycin solution, and CT26 cells were grown in RPMI 1640 supplemented with 10% fetal bovine serum and 1% penicillin-streptomycin solution. All cells were maintained at 37 ^o^C in a humidified incubator with 5% CO_2_.

**Cell uptake study**

Fluorescein isothiocyanate (FITC) as the fluorescence probe was used for cellular uptake test. SW480 cells were seeded in 96-well plates with a density of 1.2*10^5^ cells per well for 24 h at 37 ^o^C with 5% CO_2_, and then the medium (Pd-Thp-Ti_3_C_2_T_x_-FITC) was added in each well. Cell images were obtained by Echo Laboratories Revolve FL at different time point over 4 h.

**Western blotting**

The expression of Bax, Bak, Bcl-Xl, Cytoplasmic (Cyt-c) and cleaved caspase 3 was analyzed by western blotting. Briefly. SW480 cells were seeded in 6-well plates with a density of 2.5*10^6^ cells per well for 24 h. Different nanocomposites with concentration of 25 ppm were added into wells for 4 h, and then irradiated under 808 nm with 1.0 W/cm^2^ for 5 min. After 24 h incubation, the protein samples were collected, and took a certain amount of protein and run on 12% SDS-prolyacrylamide gels, and then transfer onto nitrocellulose membranes. The proteins expression was detected by protein-specific rabbit antibody and corresponding HRP-conjugated anti-rabbit IgG, then the blots were recorded through a luminol-based enhanced chemiluminescence HRP substrate.

**Mitochondrial assay**

MitoTracker Red CMXRos was used to detect the condition of mitochondria. SW480 cells were cultured in medium with different sample (25 ppm) for 4 h. The cells were irradiated under 808 nm laser at 1.0 W/cm^2^ for 5 min, and then washed with PBS for 1 time. The fresh medium containing certain amount of MitoTracker Red CMXRos were added staying for 25min, and then washed with PBS for 2 times. Next, cells were immobilized with 4% paraformaldehyde for 20 min and washed with PBS for 2 times. Last, SW480 cells were cultured with 4',6-diamidino-2-phenylindole (DAPI) in PBS for 10 min, and then washed with PBS for 3 times. Cell images were recorded by Echo Laboratories Revolve FL.

**Biosafety assay**

Twenty healthy female BALB/c mice (8 weeks old) were purchased and randomly divided into 4 groups (n=5), followed by the intravenous injection of PBS, Ti_3_C_2_T_x_ (5 mg/mL), Thp-Ti_3_C_2_T_x_ (5 mg mL^-1^) and Pd-Thp-Ti_3_C_2_T_x_ (5 mg/mL). The body weight was recorded every 2 days to analyze the physical condition of mice. At the 14^th^ day, mice were sacrificed, and the blood was collected to research the standard hematology test and serum biochemistry assy. The major organs including heart, kidney, liver, lung and spleen were stained with hematoxylin and eosin H&E to study the state of these organs.


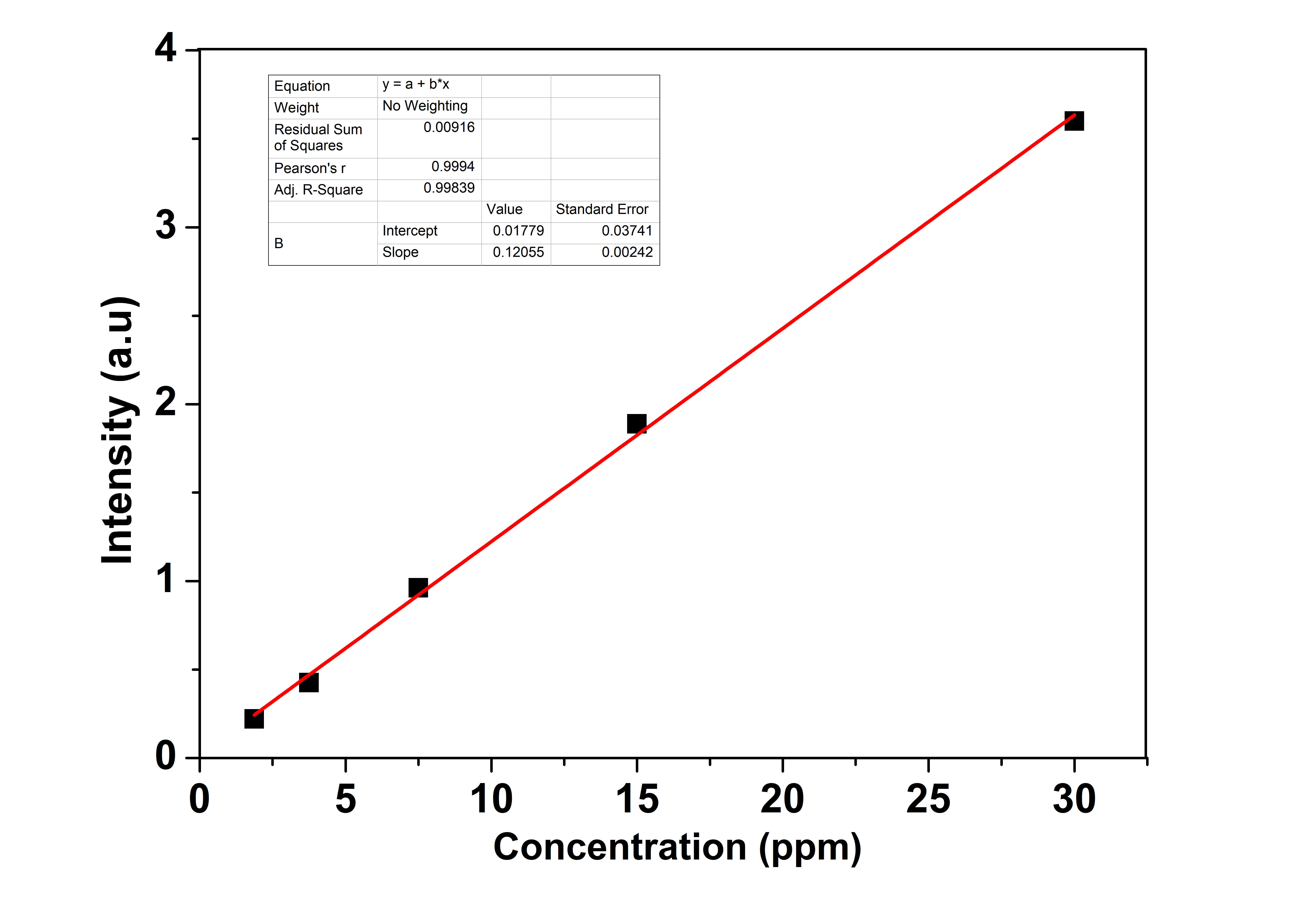


**Figure S1:** The standard curve correlating the UV-vis-NIR absorption intensity with the concentration of free Thp (R^2^=0.99).


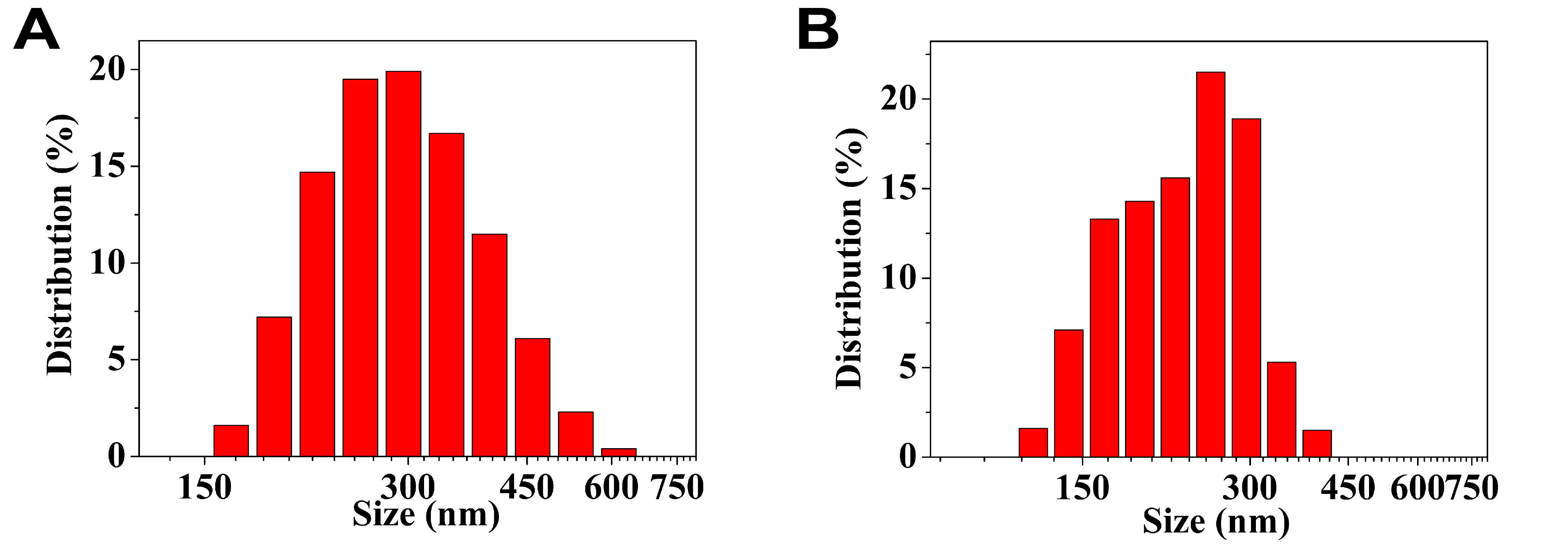


**Figure S2:** (A) Size distribution of Ti_3_C_2_T_x_ nanosheets as determined by DLS. (B) Size distribution of Pd-Thp-Ti_3_C_2_T_x_ after preparation for 8 days as determined by DLS


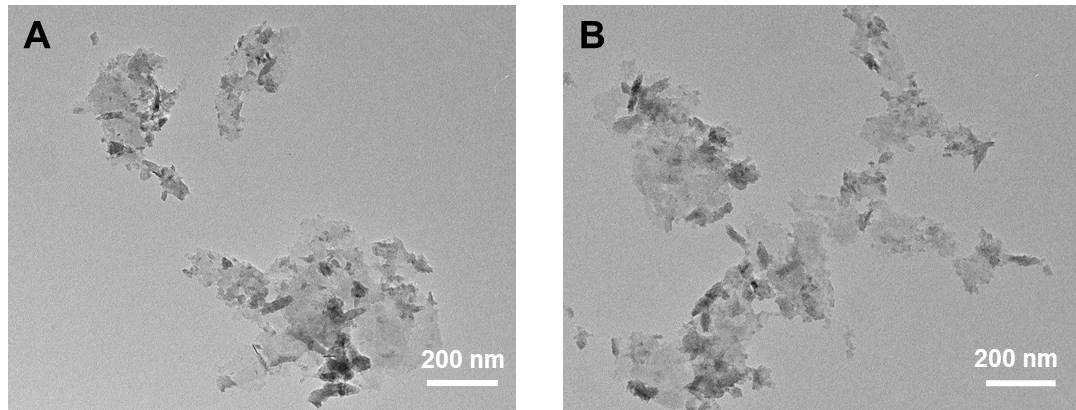


**Figure S3:** TEM images of (A) Thp-Ti_3_C_2_T_x_ and (B) Pd-Thp-Ti_3_C_2_T_x_.


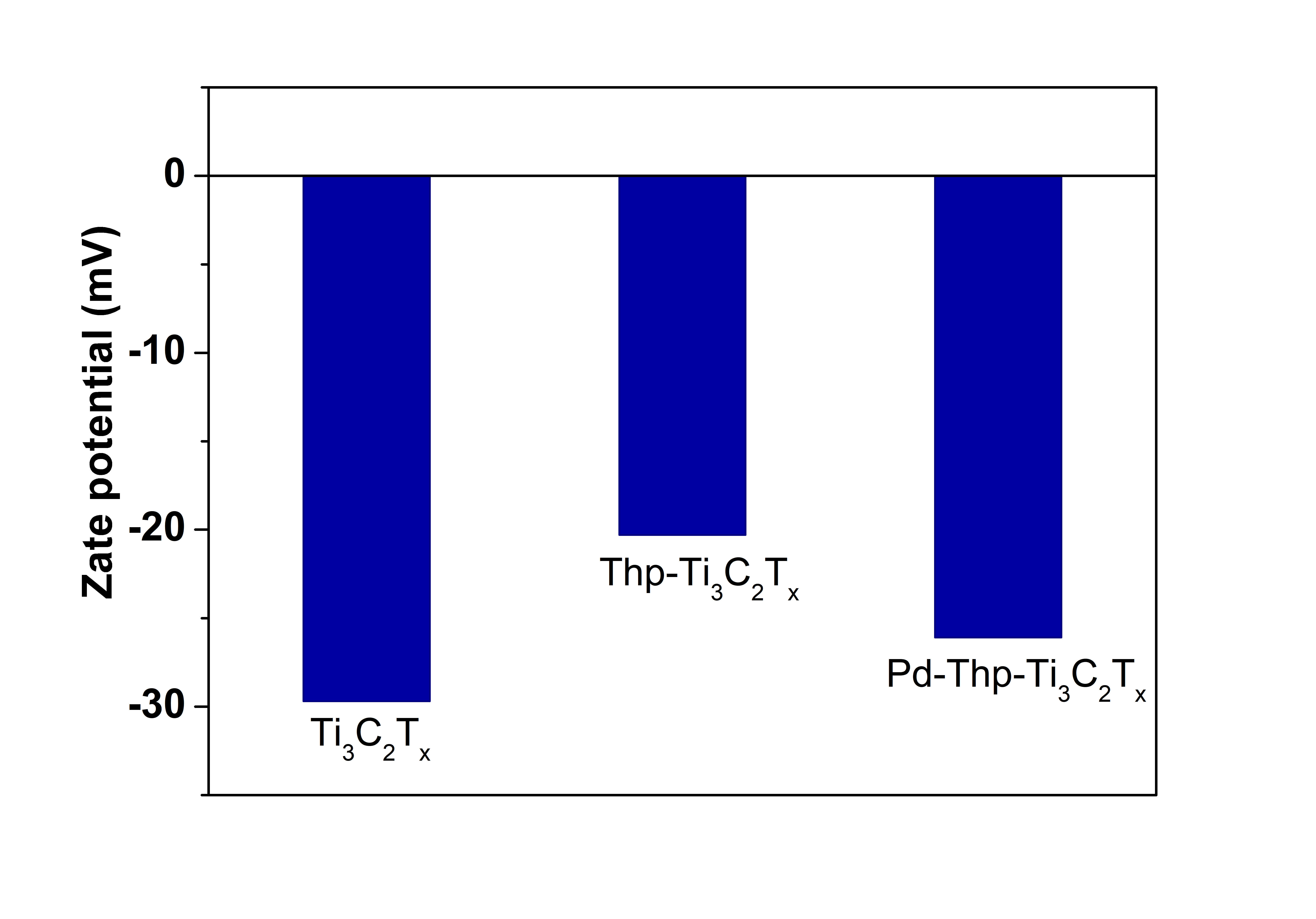


**Figure S4:** Zeta potential of Ti_3_C_2_T_x_, Thp-Ti_3_C_2_T_x_ and Pd-Thp-Ti_3_C_2_T_x_.


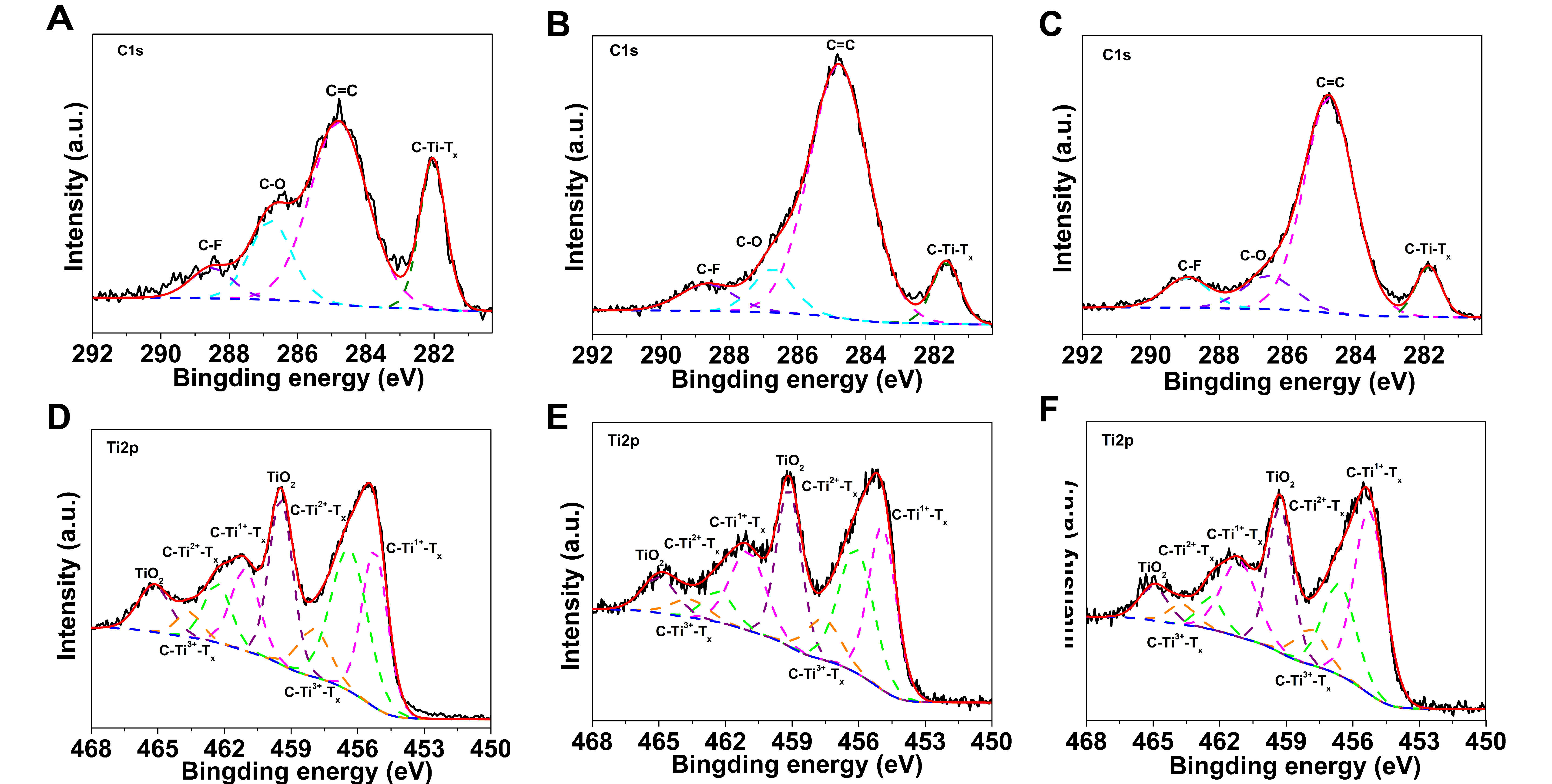


**Figure S5:**

(A) C1s and (D) Ti2p high-resolution XPS spectra of Ti_3_C_2_T_x_. (B) C1s and (E) Ti2p high-resolution XPS spectra of Thp-Ti_3_C_2_T_x_. (C) C1s and (F) Ti2p high-resolution XPS spectra of Pd-Thp-Ti_3_C_2_T_x_.

**Table S1:** Summary of XPS Ti 2p peaks of different samples

| Sample | Binding energy (eV) | | Assigned to |
| --- | --- | --- | --- |
|  | Ti 2p_3/2_ | Ti 2p_1/2_ |  |
| Ti_3_C_2_T_x_ | 455.2 | 461.1 | C-Ti^1+^-T_x_ |
|  | 456.4 | 462.3 | C-Ti^2+^-T_x_ |
|  | 457.9 | 463.6 | C-Ti^3+^-T_x_ |
|  | 459.4 | 465.2 | TiO_2_ |
| Thp-Ti_3_C_2_T_x_ | 455.0 | 461.0 | C-Ti^1+^-T_x_ |
|  | 456.1 | 462.3 | C-Ti^2+^-T_x_ |
|  | 457.6 | 463.6 | C-Ti^3+^-T_x_ |
|  | 459.1 | 465.0 | TiO_2_ |
| Pd-Thp-Ti_3_C_2_T_x_ | 455.2 | 461.1 | C-Ti^1+^-T_x_ |
|  | 456.5 | 462.4 | C-Ti^2+^-T_x_ |
|  | 457.9 | 463.7 | C-Ti^3+^-T_x_ |
|  | 459.3 | 465.0 | TiO_2_ |


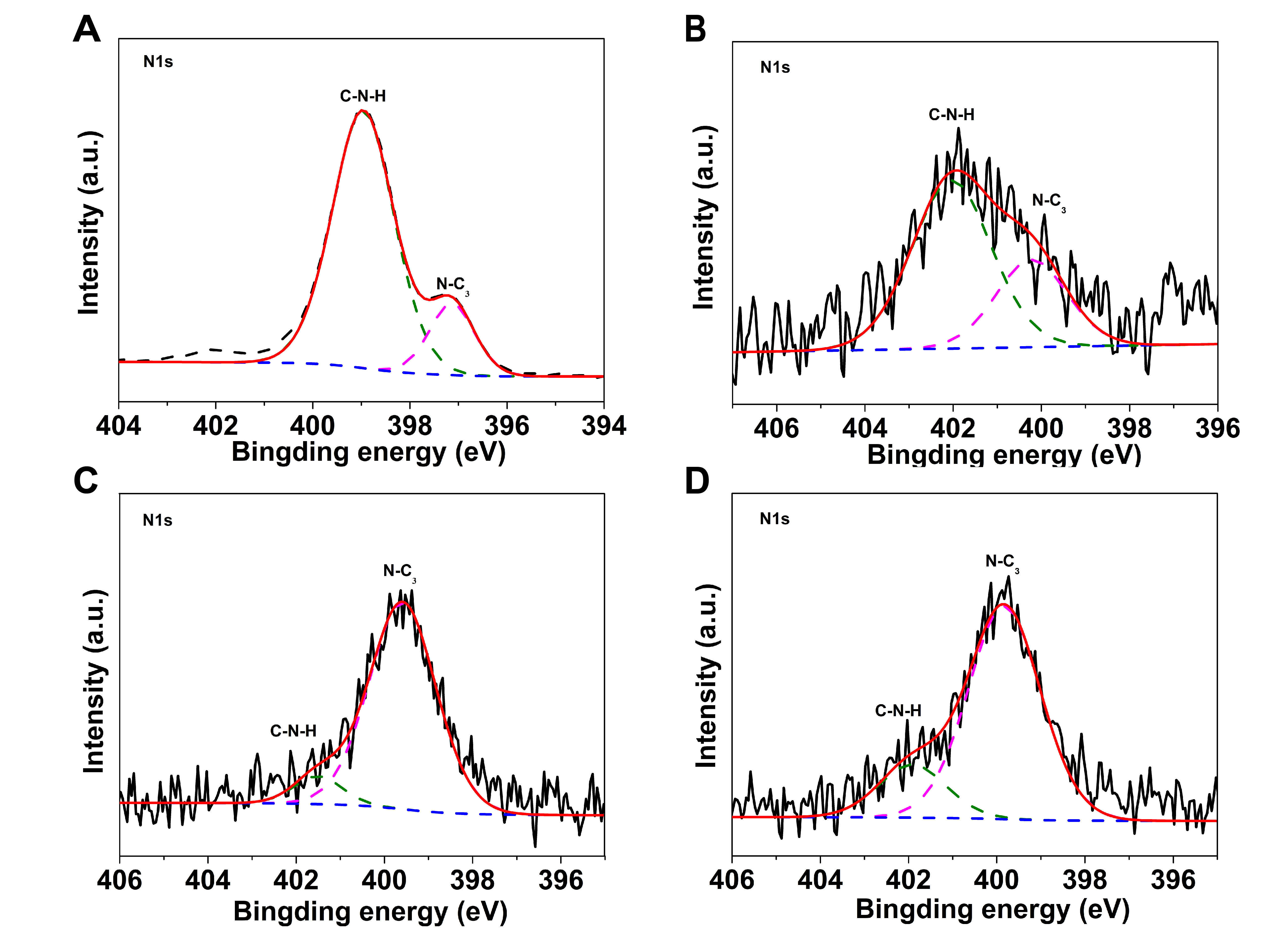


**Figure S6:** N1s high-resolution XPS spectra of (A) Thp, (B) Ti_3_C_2_T_x_, (C)Thp-Ti_3_C_2_T_x_ and (D) Pd-Thp-Ti_3_C_2_T_x_.

**Table S2** Summary of XPS N1s peaks of different samples

| Sample | Binding energy (eV) | Assigned to |
| --- | --- | --- |
| Thp | 397.2 | N-C_3_ |
|  | 399.0 | C-N-H |
| Ti_3_C_2_T_x_ | 400.2 | N-C_3_ |
|  | 402.0 | C-N-H |
| Thp-Ti_3_C_2_T_x_ | 399.6 | N-C_3_ |
|  | 401,5 | C-N-H |
| Pd-Thp-Ti_3_C_2_T_x_ | 399.8 | N-C_3_ |
|  | 401.9 | C-N-H |


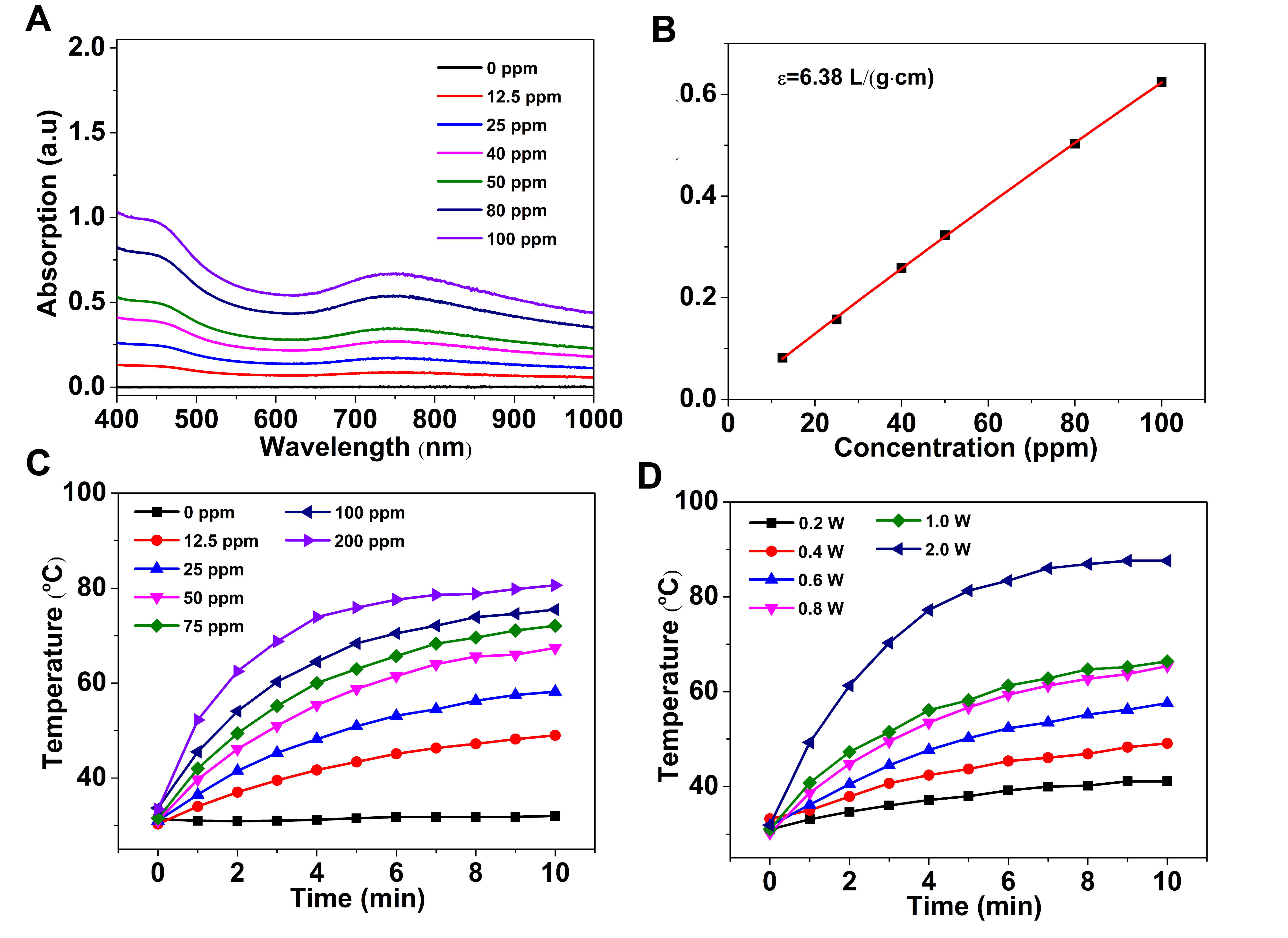


**Figure S7:**

(A) UV−vis−NIR spectra of Pd-Thp-Ti_3_C_2_T_x_ nanosheet dispersed in deionized water with certain concentrations (0, 12.5, 25, 40, 50, and 100 ppm) and (B) corresponding normalized absorbance intensity at λ=808 nm divided by the characteristic length of the cell (A/L) at elevated concentrations (R^2^=0.99). Photothermal-heating curves of Pd-Thp-Ti_3_C_2_T_x_ at (C) different concentration (808 nm, 1.0 W/cm^2^, 10 min) and (D) power density under 808 nm (50 ppm).


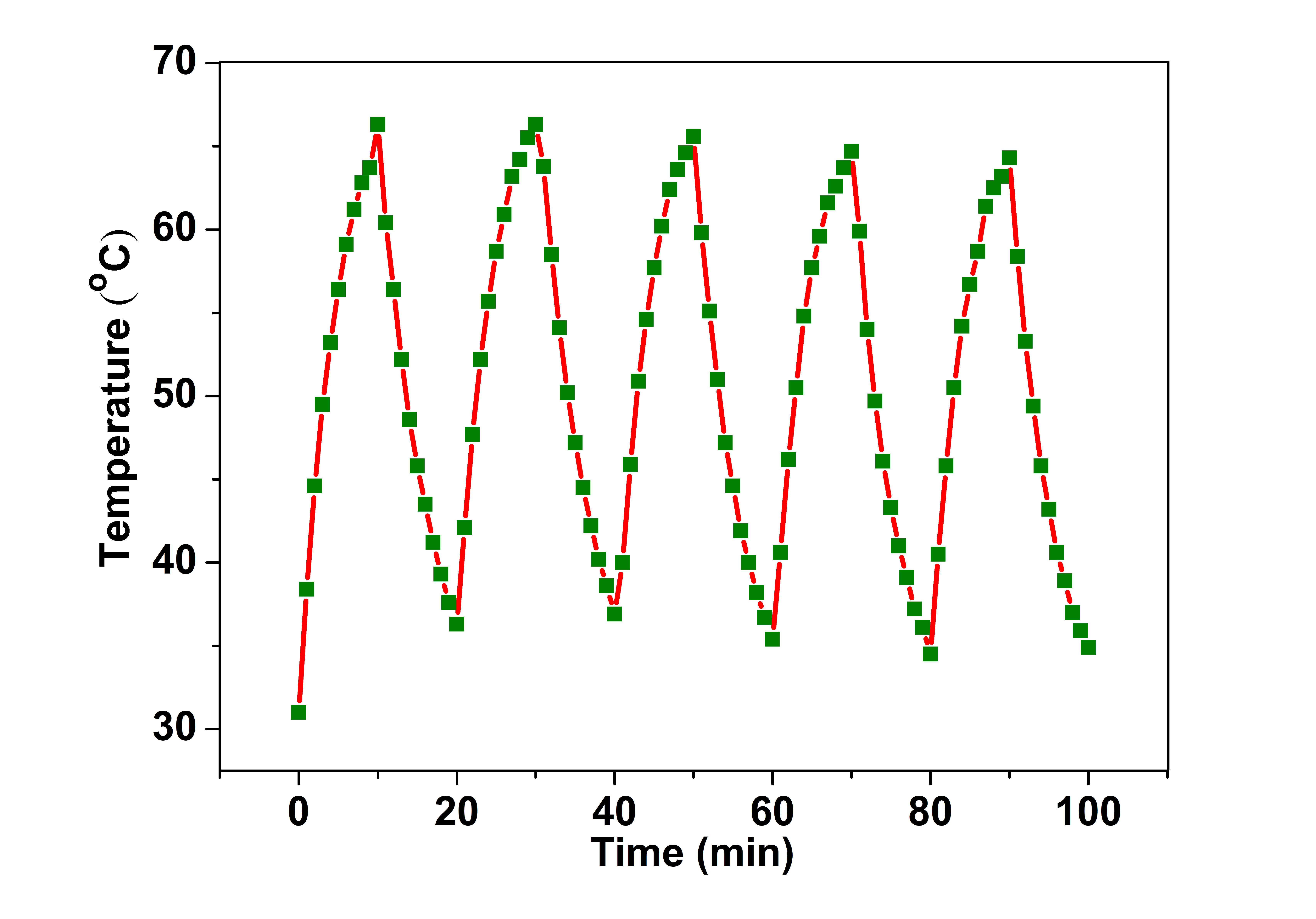


**Figure S8:** Recycling-heating curves of Pd-Thp-Ti_3_C_2_T_x_ (50 ppm) exposed to 808 nm at 1.0 W/cm^2^ for five laser on/off cycles.


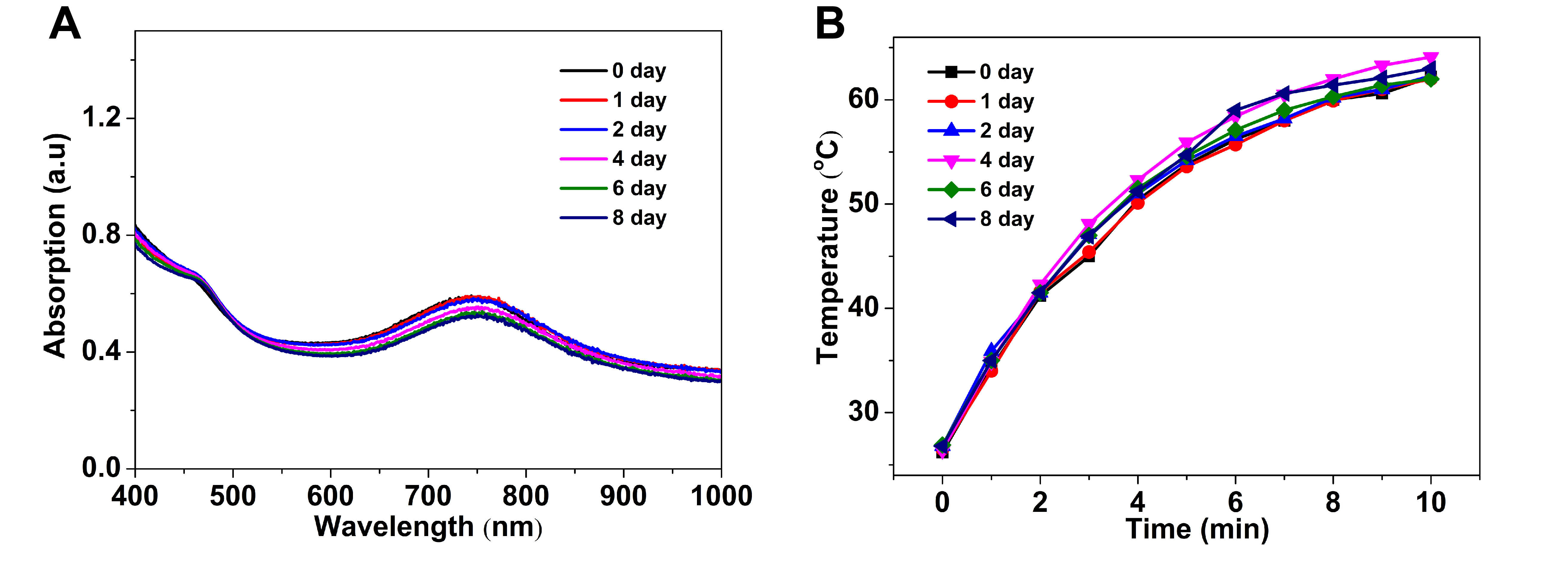


**Figure S9:**

(A) UV−vis−NIR spectra of Pd-Thp-Ti_3_C_2_T_x_ at different time (50 ppm). (B) Photothermal-heating curves of Pd-Thp-Ti_3_C_2_T_x_ at different time (808 nm, 1.0 W/cm^2^ and 50 ppm).


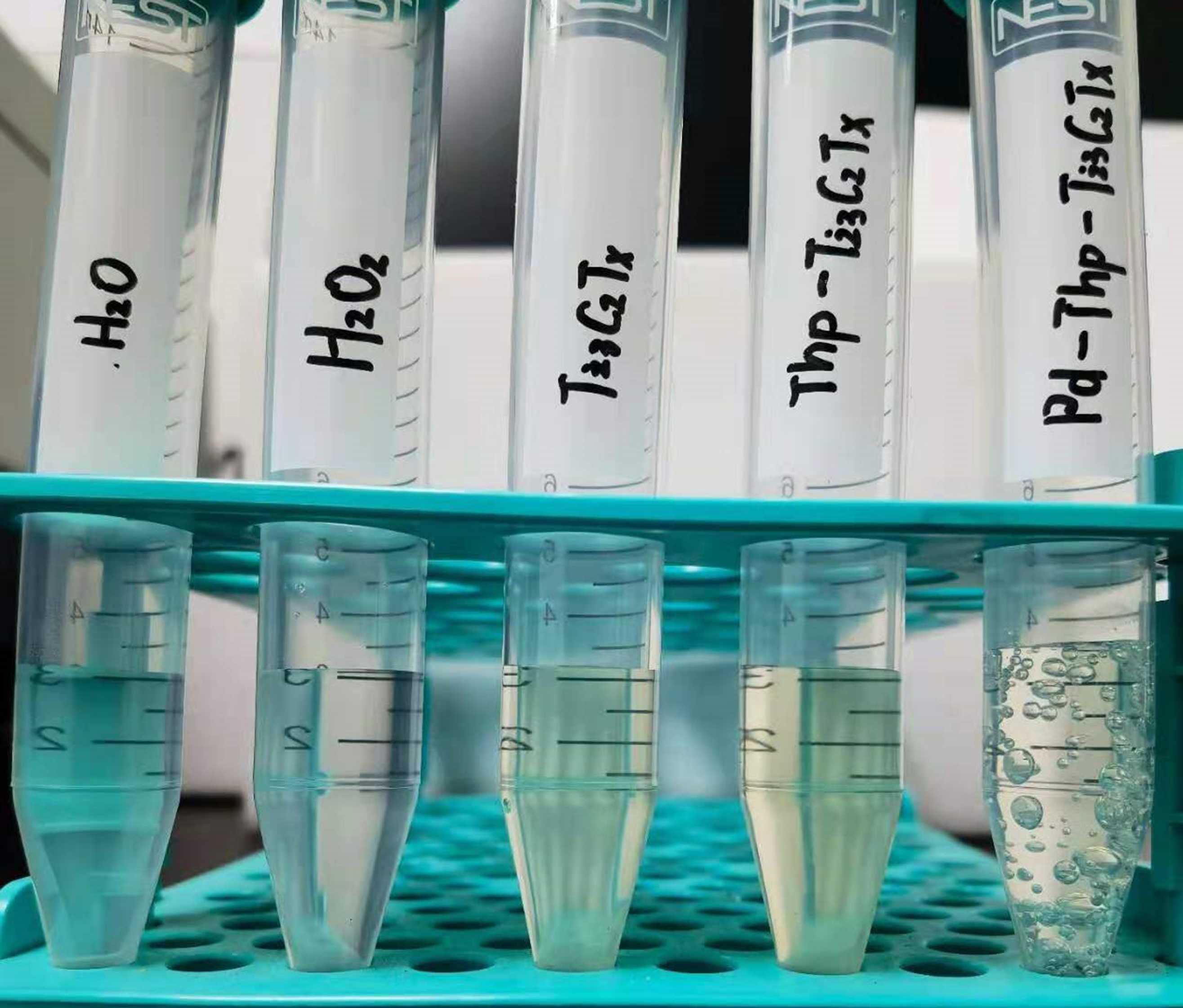


**Figure S10:** Bubbles generation in pure H_2_O, H_2_O_2_, Ti_3_C_2_T_x_, Thp-Ti_3_C_2_T_x_ and Pd-Thp-Ti_3_C_2_T_x_ aqueous solution.


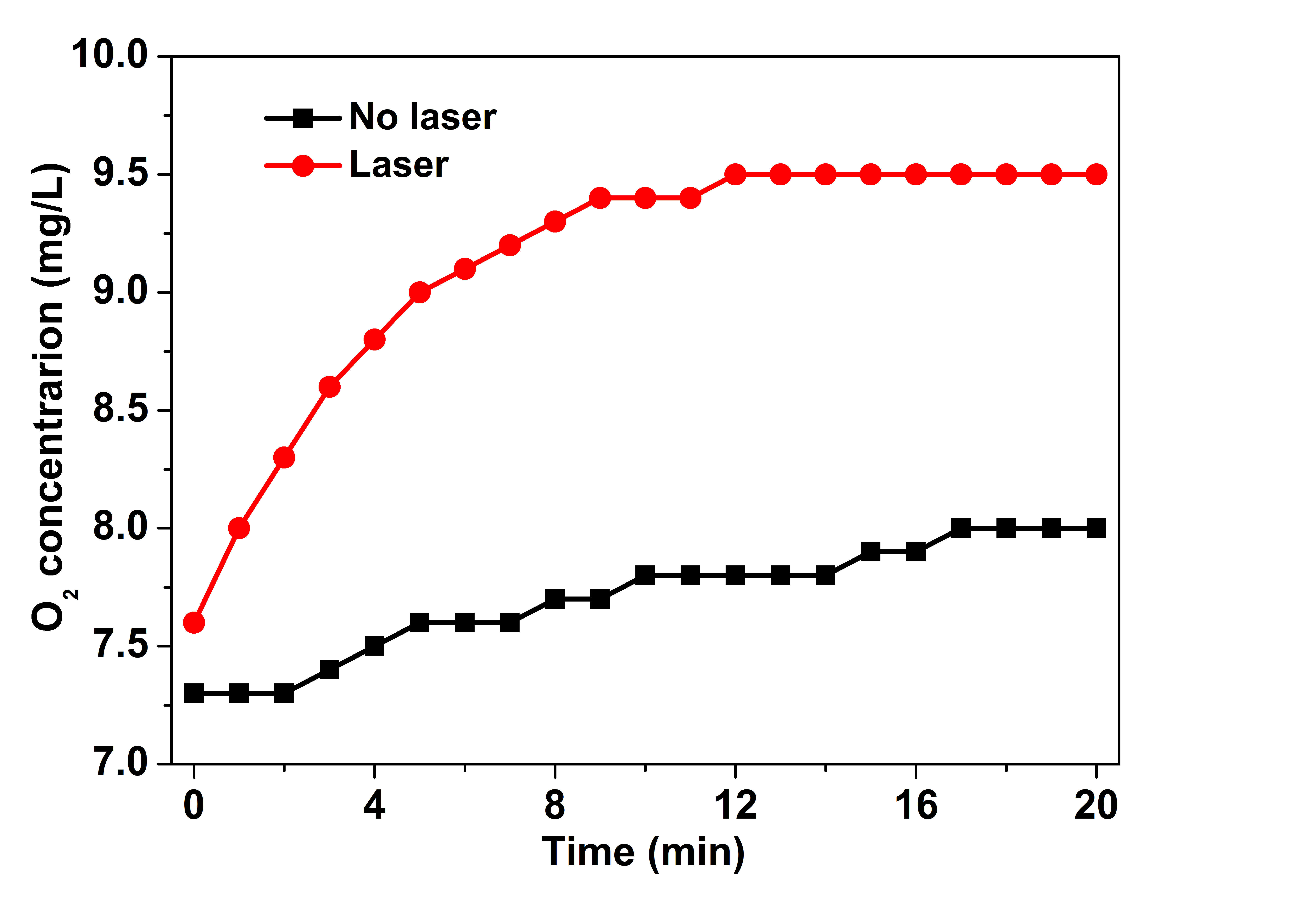


**Figure S11:** O_2_ concentration of H_2_O_2_ solution in dark or under 808 nm irradiation at 1.0 W/cm^2^, in which the concentration of H_2_O_2_ was the same as that tested in Fig. 3(d-e).


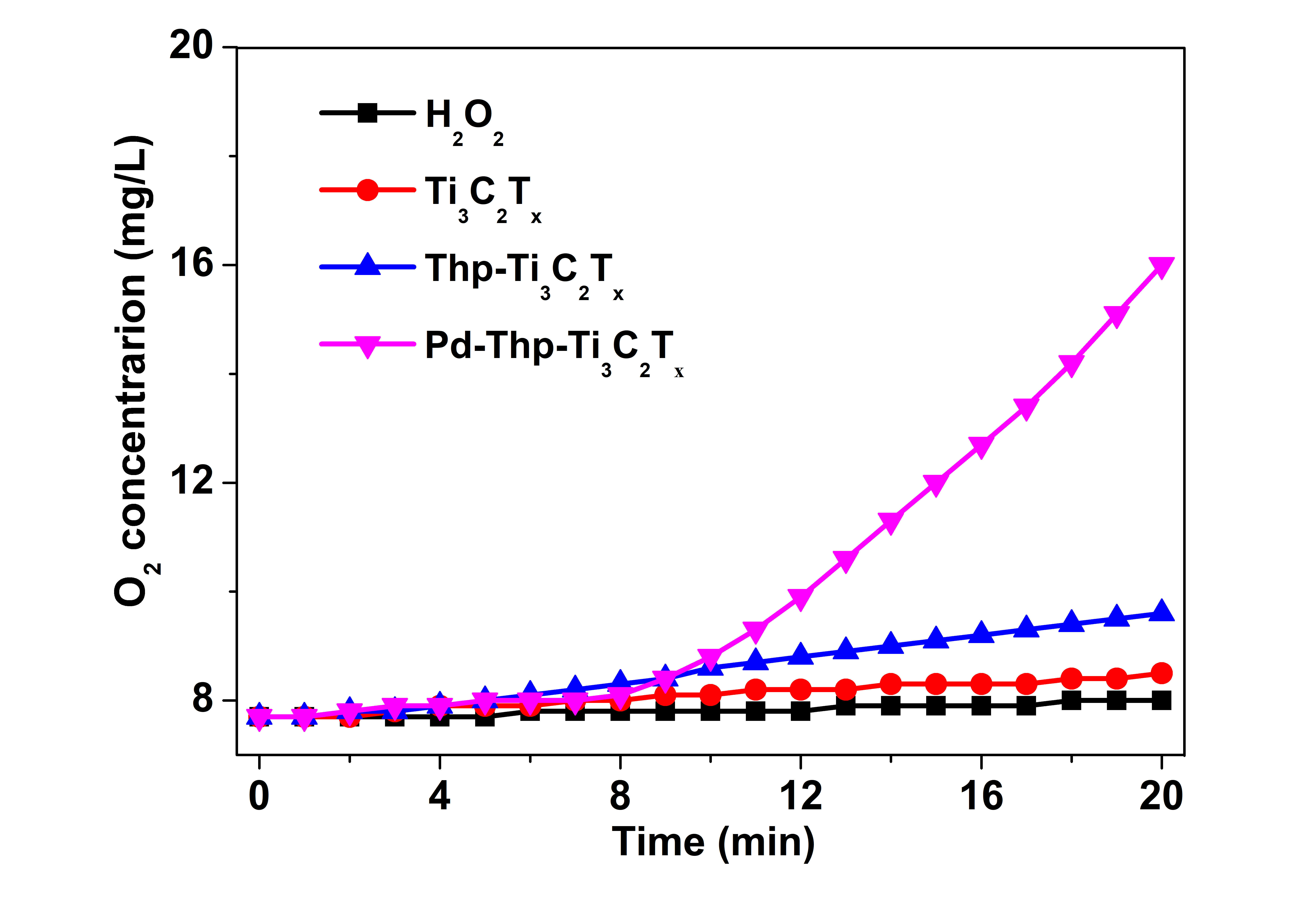


**Figure S12:** O_2_ concentration of H_2_O_2_ solution after adding different nanocomposites without irradiation.


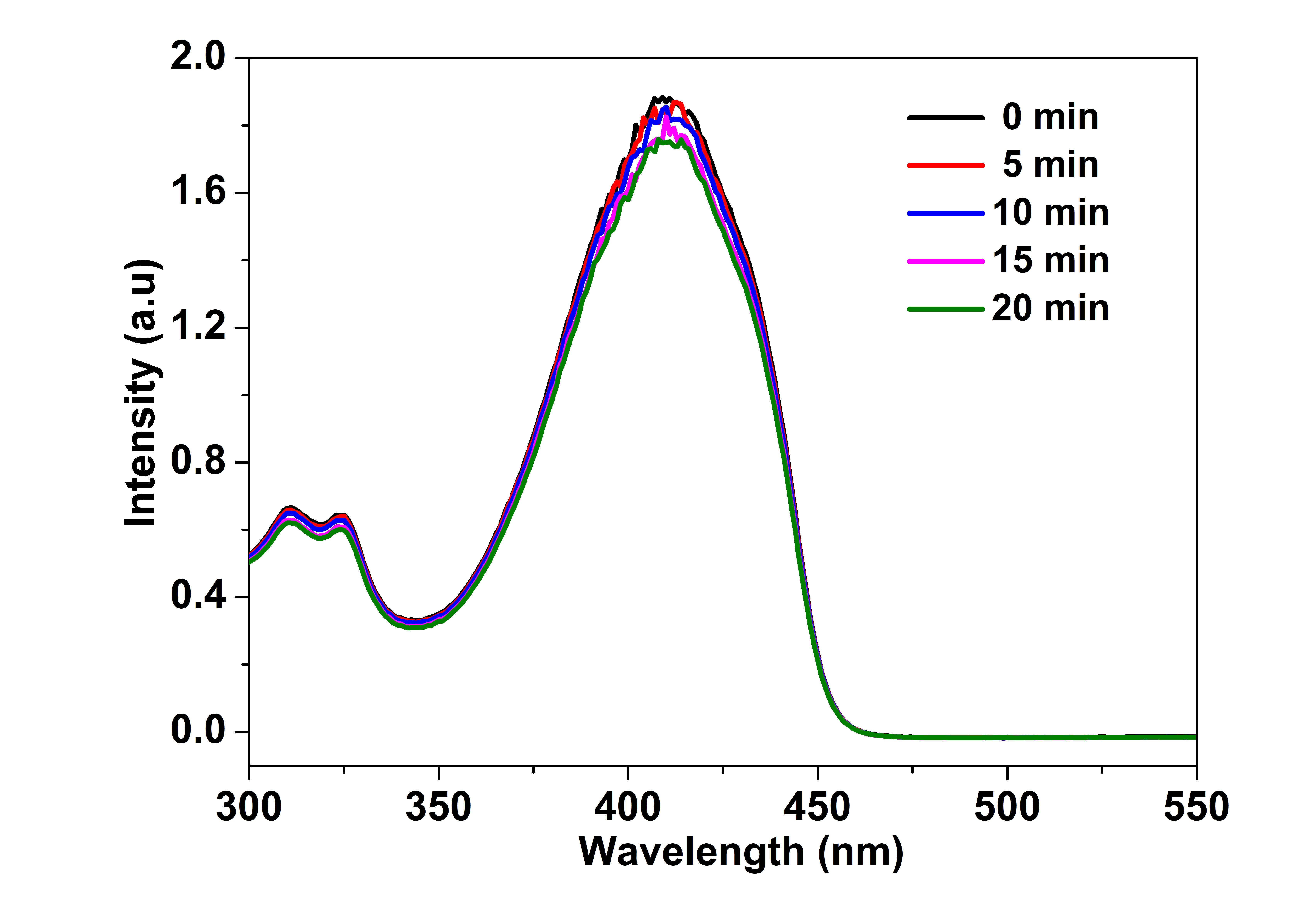


**Figure S13:** UV-vis-NIR absorption spectra of DPBF under laser irradiation (808 nm, 1.0 W/cm^2^) for different duration.


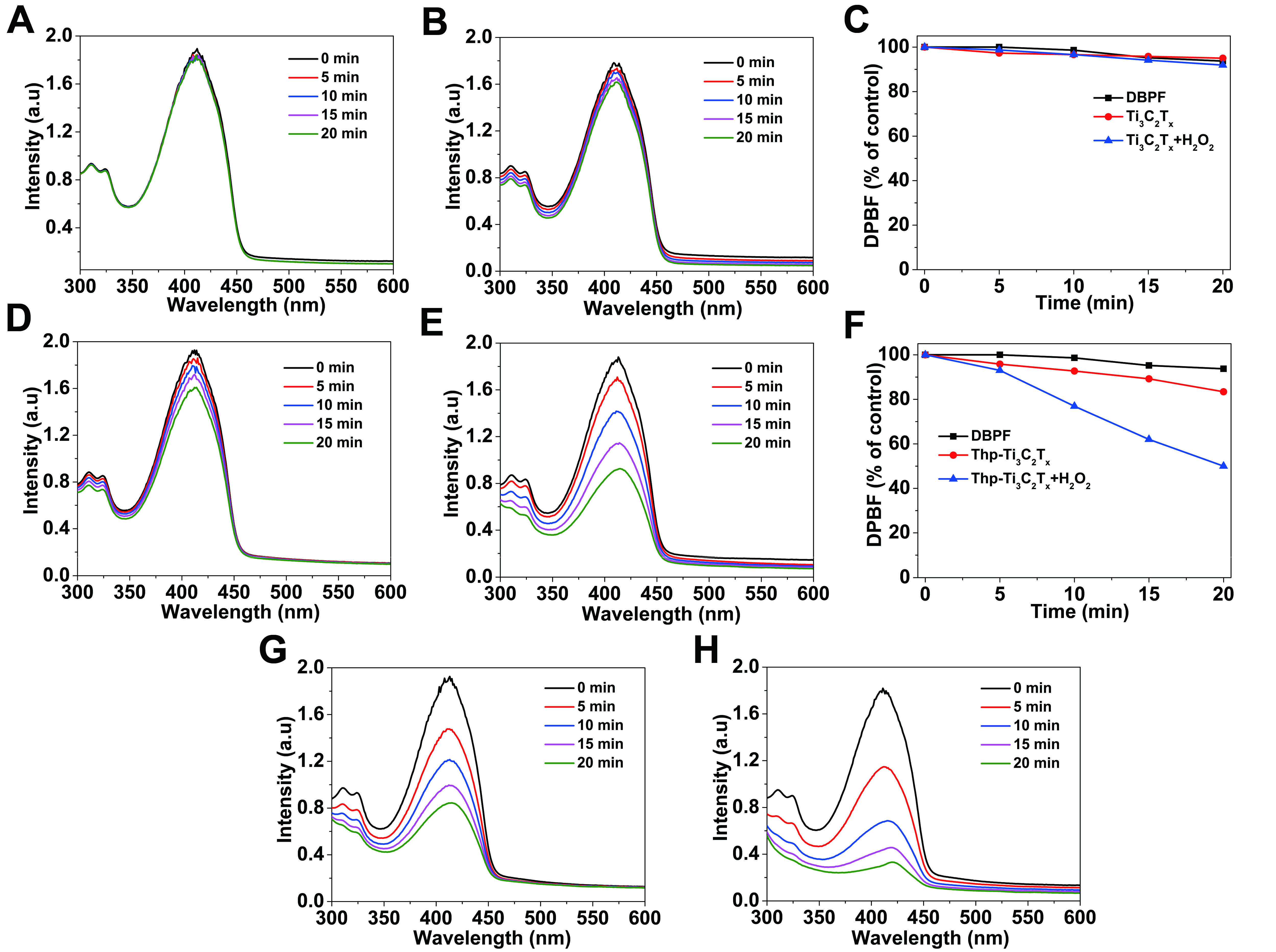


**Figure S14:**

Absorption spectrum changes of DPBF under 808 nm (1.0 W/cm^2^) laser irradiation with Ti_3_C_2_T_x_ in the (A) absence or (B) presence of H_2_O_2_. Decay curves of DPBF absorption at 410 nm in (C) Ti_3_C_2_T_x_ and (F) Thp-Ti_3_C_2_T_x_ with or without H_2_O_2_ after different time point of irradiation (808 nm, 1.0 W/cm^2^). Absorption spectrum changes of DPBF under 808 nm (1.0 W/cm^2^) laser irradiation with Thp-Ti_3_C_2_T_x_ in the (D) absence or (E) presence of H_2_O_2_. Absorption spectrum changes of DPBF under 808 nm (1.0 W/cm^2^) laser irradiation with Pd-Thp-Ti_3_C_2_T_x_ in the (G) absence or (H) presence of H_2_O_2_.


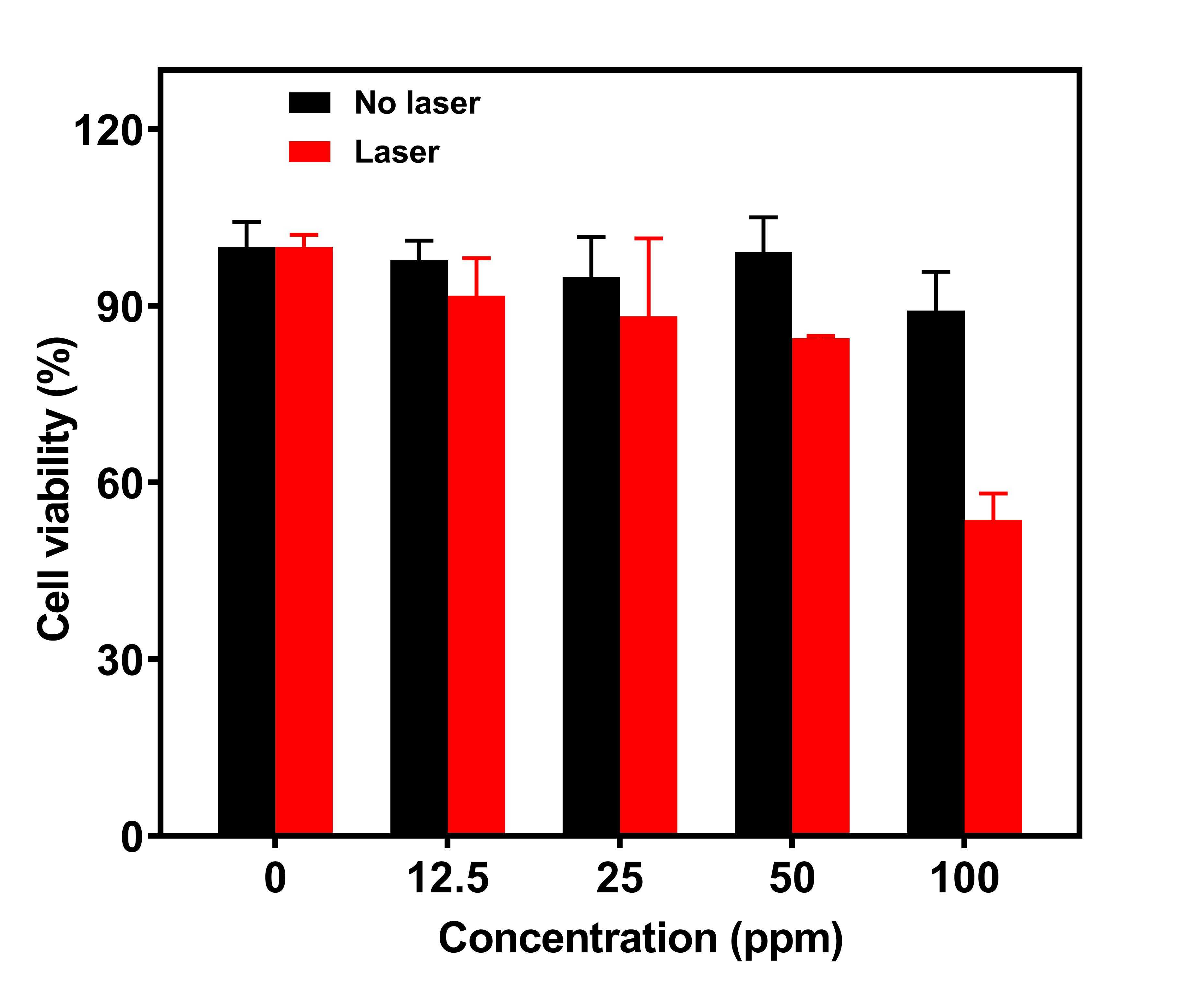


**Figure S15:** Cell viabilities of SW480 cells incubated with free Thp at different concentration, in which the concentration of Thp was corrected the same as that of Thp-Ti_3_C_2_T_x_ and Pd-Thp-Ti_3_C_2_T_x_.


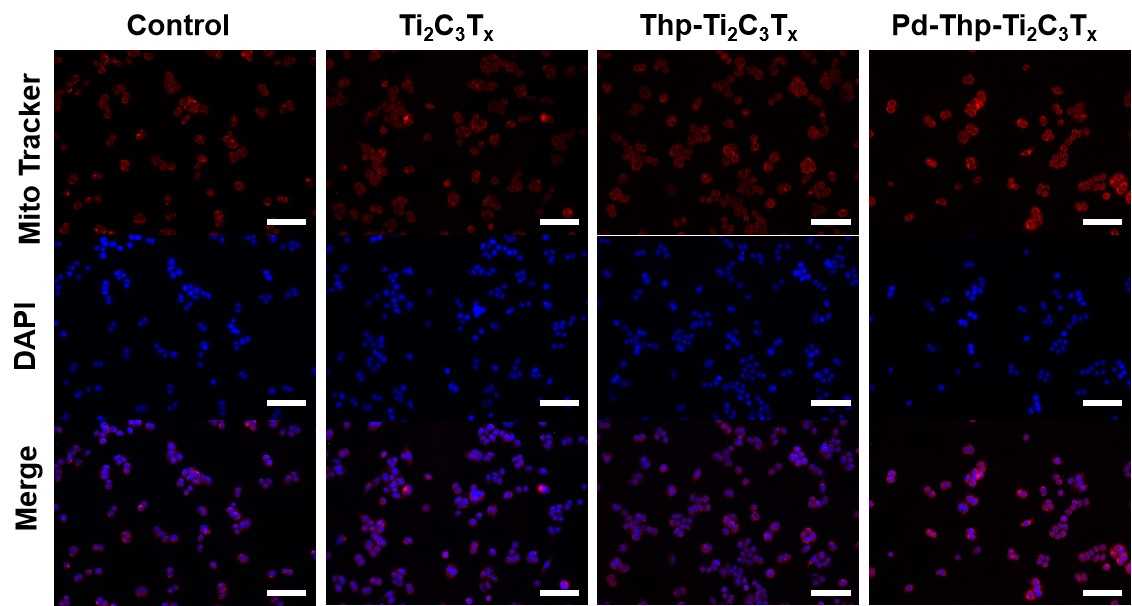


**Figure S16:** Cells treated with different composites were stained with MitoTracker Red CMXRos without irradiation. (Treatment condition: 12.5 ppm, 808 nm, 1.0 W /cm^2^, 5 min; scale bar: 100 μm).


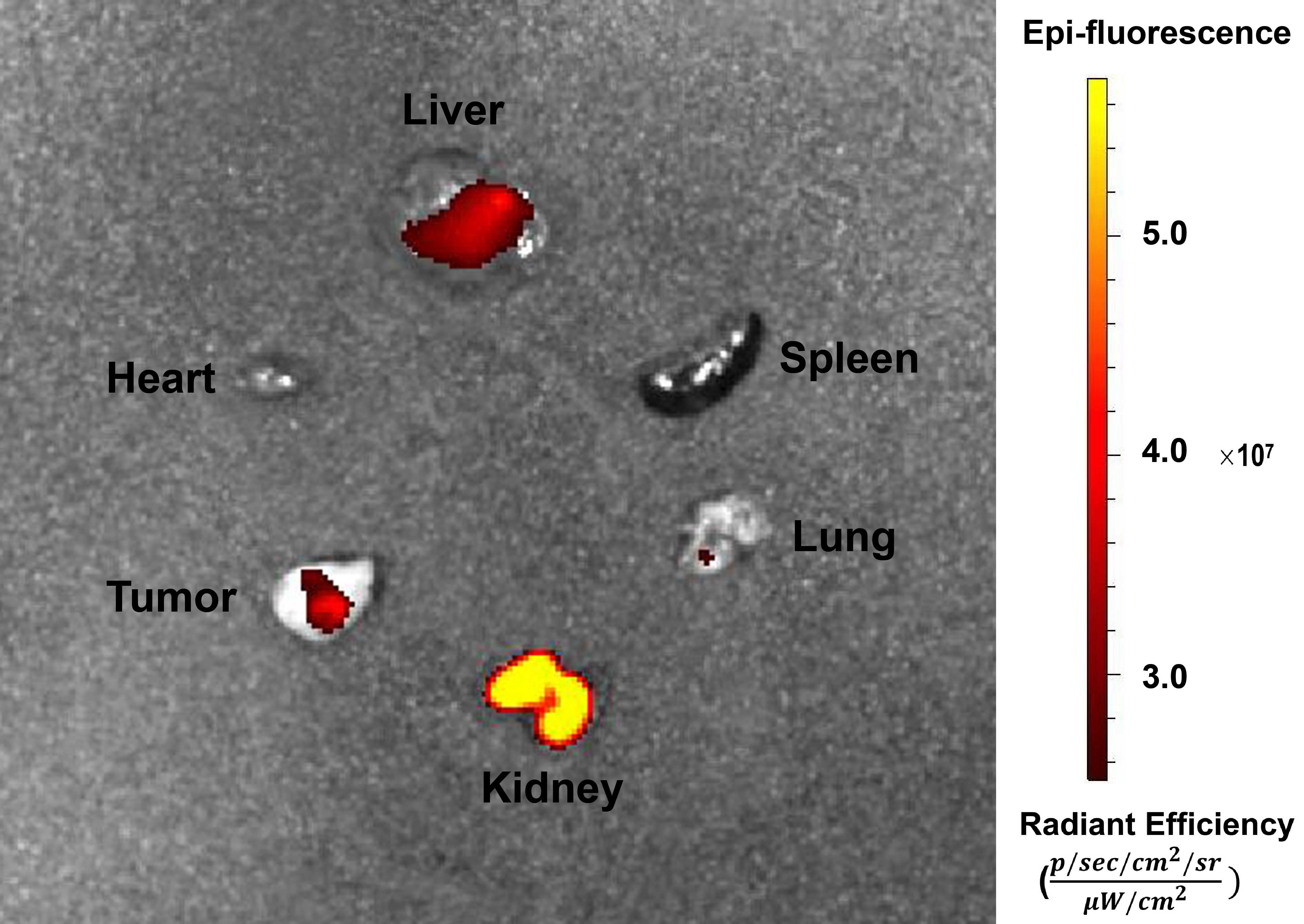


**Figure S17:** Fluorescence images of major organs and the tumors of mice 72 h post-irradiation.


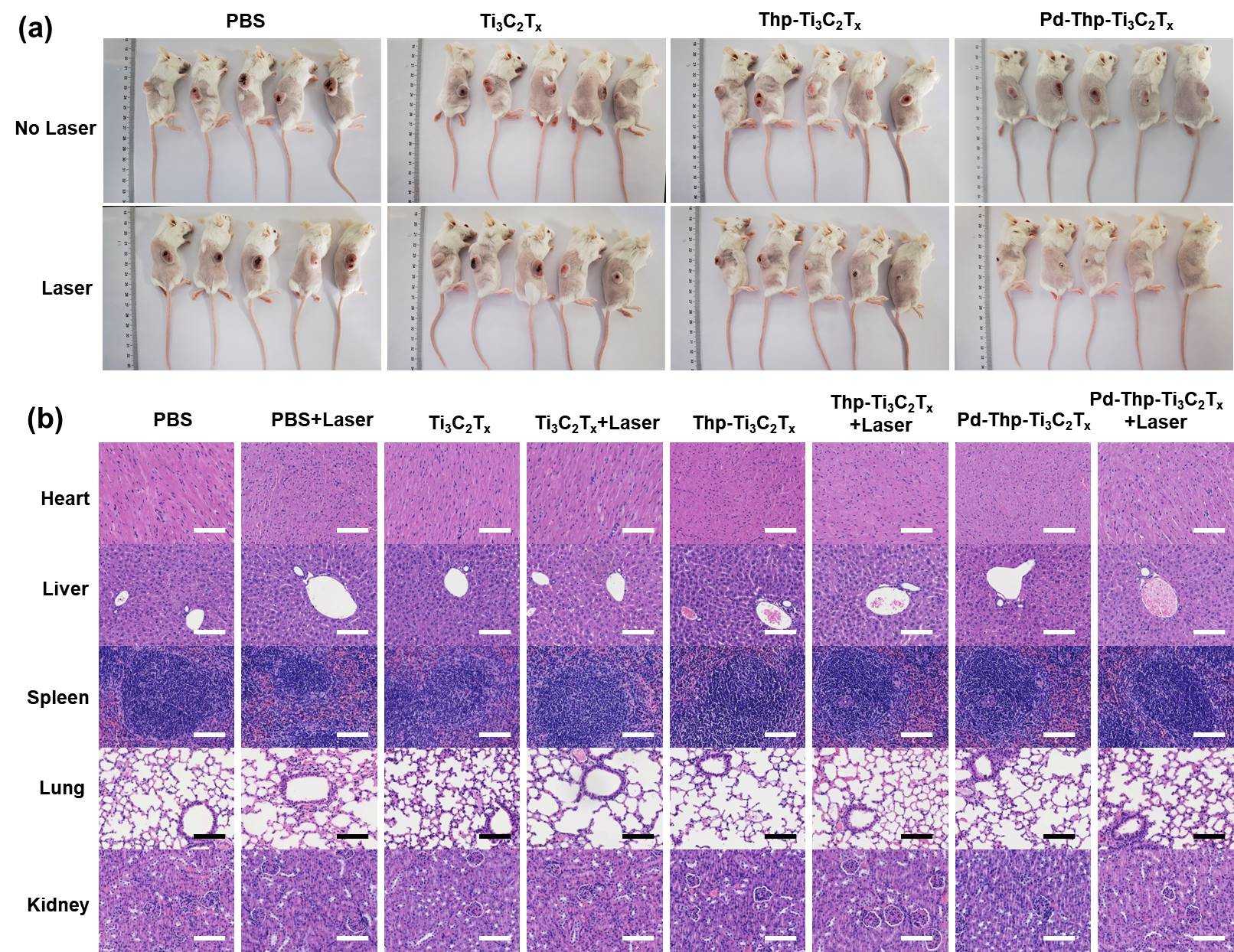


**Figure S18:**

(A) Digital images of each CT26-tumor-bearing mice of all groups after different treatments at 14^th^ day. (B) H&E-stained tissue sections of major organs from BALB/c mice in biosafety experiments (Scale bar: 100 µm).
